# Immune, RNA, and Neurocognitive Genetic Networks in Bipolar Disorder Subtypes: A Transcriptomic Meta-Analysis

**DOI:** 10.21203/rs.3.rs-3508951/v1

**Published:** 2024-01-17

**Authors:** Tyler Jang, Marcus Kaul

**Affiliations:** 1 University of California, Riverside, Graduate Program of Genetics, Genomics, and Bioinformatics, Riverside, 92507, USA; 2 University of California, Riverside, Department of Biomedical Sciences, Riverside, 92507, USA

## Abstract

**Background:**

Little is known about the pathogenesis of Bipolar Disorder, and even less is known about the genetic differences between its subtypes. Bipolar Disorder is classified into different subtypes, which present different symptoms and lifetime courses. While genetic studies have been conducted in Bipolar Disorder, most examined the gene expression of only Bipolar Disorder Type 1. Studies that include Bipolar Disorder Type 1 and Bipolar Disorder Type 2 often fail to differentiate them into separate conditions. Few large transcriptomic meta-analyses in Bipolar Disorder have been conducted to identify genetic pathways. Thus, using publicly available data sets we aim here to uncover significant differential gene expression that allows distinguishing Type 1 and Type 2 Bipolar Disorders, as well as find patterns in Bipolar Disorder as a whole.

**Methods:**

We analyze 17 different gene expression data sets from different tissue in Bipolar Disorder using GEO2R and manual analysis, of which 15 contained significant differential gene expression results. We use STRING and Cytoscape to examine Gene Ontology to find significantly affected genetic pathways. We identify hub genes using cytoHubba, a plugin in Cytoscape. We find genes common to data sets of the same material or subtype.

**Results:**

12 out of 15 data sets are enriched for immune system and RNA related pathways. 9 out of 15 data sets are enriched for neurocognitive and metal ion related GO terms. Analysis of Bipolar Disorder Type 1 vs Bipolar Disorder Type 2 revealed most differentially expressed genes were related to immune function, especially cytokines. Terms related to synaptic signaling and neurotransmitter secretion were found in down-regulated GO terms while terms related to neuron apoptosis and death were up-regulated. We identify the gene SNCA as a potential biomarker for overall Bipolar Disorder diagnosis due to its prevalence in our data sets.

**Conclusions:**

The immune system and RNA related pathways are significantly enriched across the Bipolar Disorder data sets. The role of these pathways is likely more critically important to the function of Bipolar Disorder than currently understood. Further studies should clearly label the subtype of Bipolar Disorder used in their research and more effort needs to be undertaken to collect samples from Cyclothymic Disorder and Bipolar Disorder Type 2.

## Introduction

Bipolar Disorder (BD) is a psychiatric disorder that has severe effects on the quality of life of those affected by the disorder. The prevalence of BD is roughly around 1 percent of the general population^1^. Rates of mortality are significantly worse for those living with BD^2^. A barrier to treatment of BD is successful and early diagnosis, which is currently limited by the subjective nature of interviews and review of medical history due to inter-rater variability^3, 4^. This leads to frequent misdiagnosis. The consequences of misdiagnosis and subsequent mistreatment with antidepressants or other medication can induce manic episodes or mood fluctuations. A study reports that 37% of individuals with BD first receive a misdiagnosis of unipolar depression, also known as major depressive disorder (MDD), from medical health professionals following their first manic or hypomanic episode^5^. This results in 23% of patients developing new or worse rapid cycling courses following treatment by antidepressants^5^.

BD is classified into different subtypes: Type 1, Type 2, Cyclothymic Disorder, and other unspecified types (BD NOS). Bipolar Disorder Type 1 (BD 1) is characterized by the experience of at least 1 manic episode which is defined by meeting 5 out of 9 criteria defined in the DSM^6^. Bipolar Disorder Type 2 (BD 2) is characterized by experiencing at least 1 hypomanic episode and 1 depressive episode. Due to this criteria, the identification of manic versus hypomanic episodes is key, but since these episodes do not always result in individuals seeking clinical treatment, depressive symptoms are more likely to be identified first^7^. Cyclothymic disorder, also known as Cyclothymia, is characterized by frequent changes in mood between hypomanic and depressive episodes. While these episodes are typically less intense as compared to BD 1 and BD 2, the episodes tend to oscillate more frequently^8^.

Thus, the wider research community has looked into the genetic underpinnings of BD. Genome-wide association studies (GWAS) analysis from Bipolar Disorder Type 1 and Bipolar Disorder Type 2 are highly genetically correlated: r=0.78, r=0.85, and r=.89^9–11^. These studies show that the subtypes belong to a joint classification as BD, but have distinct genetic differences. For example, Charney et al. identify polygenic risk alleles related to schizophrenia (SZ) as being associated more with BD 1 than BD 2^9^. However, it is currently not well understood what genes are responsible for the genetic difference.

Some studies have attempted to identify genetic differences between the subtypes of BD. A 2020 review paper on genetic differences between BD 1 and BD 2 found a total of 47 papers analyzing patients with BD 2 specifically^7^. Of this, 39 papers were candidate gene studies and the other 8 were GWAS. This study identified that the main genetic differences were in genes related to the monoaminergic system and neuroplasticity. They additionally report that TNF-308 G/A and IFN-*γ* +874 T/A polymorphisms were associated only with BD 2. Candidate gene studies have identified singular gene expression differences between BD 1 and BD 2. For example, methylation of BDNF was reported to be increased in BD 2 but not in BD 1^12^. However, this information seems insufficient to identify the difference in pathogenesis between BD 1 and BD 2.

Notably, there are zero genetic studies on the BD subtype Cyclothymia. This is despite Cyclothymia having a high prevalence and being part of the Diagnostic and Statistical Manual of Mental Disorders (DSM) since the DSM-II^8, 13,14^. Some studies even indicate that it may be the most prevalent form of BD of all subtypes^8^. The few studies that look at Cyclothymia do so in the context of cyclothymic temperament and even those are few, limited to studies about the gene expression of a handful of genes^15^.

Structural differences between BD 1 and BD 2 have been identified by neuroimaging. Abnormalities of both subtypes tend to be located primarily in the frontal lobe^16^. however, BD 1 abnormalities were more lateralized to the right hemisphere while BD 2 abnormalities were bilaterally distributed more towards the left temporal lobe^16^. Specifically, both subtypes of BD displayed fiber impairments in the thalamus, anterior cingulate, and inferior frontal areas. However, BD 2 showed more fiber impairments in the temporal and inferior prefrontal regions^16^. Further studies show that white matter volume differs between subtypes, where BD 1 has less volume than BD 2^17^. Given this information, analysis of gene expression from brain tissue from the frontal lobe tissue could be anticipated to provide the most relevant insights.

A review of the literature by Pinto et al. implicates the importance of glial cells in BD^18^. Patients with BD show a reduced number of glial cells in the brain^19^. In vivo neuroinflammation has been reported in the hippocampus in individuals with BD^20^. Abnormal function in microglia and astrocytes have been implicated, such that in individuals with BD, greater pro-inflammatory microglial activation has been found compared to controls^18^. An in vitro model using induced pluripotent stem cells (iPSCs) from BD patients demonstrated that when BD astrocytes are co-cultured with neurons, neuronal activity is reduced^21^. The same study also identifies that IL-1*β* stimulates BD astrocytes to increase the secretion of IL-6. When partially blocking IL-6 with an antibody, neuronal activity was partially rescued^21^. These results suggest that glial cells potentially play a larger role in BD than is currently appreciated.

Across the literature, the expression of cytokines is found at higher levels in patients with BD compared to controls. Ghafouri-Fard et al. found higher expression of IL-1*β*, IL-10, IFN-*γ*, TNF-*α*, TGF-*β*, and IL-2 in male patients with BD versus controls^22^. Meta-analysis of blood cytokine alterations in patients with psychiatric disorders showed that IL-6, TNF-*α*, sIL-2R, and IL1RA were significantly increased in BD patients in acute mania compared to controls^23^. The same study also showed that euthymic patients with BD have small elevations in IL-10, IL-6, IL-1*β*, and sTNF-R1 and medium to large elevations of IL-4, sIL-6R, and sIL-2R. In patients with BD 1, genes related to cytokine-cytokine receptor interactions were found to be enriched in manic episodes^24^. Bai et al. observe significant differences in cytokine expression for sTNF-R1 between BD 1 and BD 2^25^. RNA-seq experiments of iPSCs generating cerebral organoids show downregulation of pathways related to cell adhesion, neurodevelopment, and synaptic biology, while also seeing an upregulation in genes involved in immune signaling^26^. Barbosa et al. identify higher levels of CCL11, CCL24, and CXCL10 and decreased levels of CXCL8 in blood samples taken from patients with BD versus controls^27^. A review article identifies elevated levels of IL4, TNF-*α*, sIL-2R, IL-1*β*, IL-6, sTNFR1, and CRP in BD patients versus controls^28^. Additionally, the same article reports that elevated levels of inflammation related markers YKL40, IL-6, sCD40L, IL1RN, CRP, and TNF-*α* are associated with poorer cognitive function in people living with BD^28^. Overall, the literature suggests elevated levels of pro-inflammatory cytokines in BD.

Due to the influence of numerous factors, a more comprehensive analysis of genetic data in BD needs to be conducted. No paper has performed a large bioinformatics comparison of RNA-seq gene expression of different BD and control data sets and none have done so for BD 1 vs BD 2. Some very recent bioinformatics studies have analyzed a limited number of RNA-Seq data sets and looked at differentially expressed genes (DEGs). Their results identify some key pathways through Gene Ontology (GO) term analysis, but they only analyze a few data sets and focus on different research questions^29–31^. Additionally, analysis needs to be performed to account for confounding factors. For example, Krebs et al. identify the need for accounting for lithium treatment in transcriptome analysis of BD^32^. These current bioinformatic studies do not contain samples from non-medicated patients in their analysis. Additionally, the identification of biomarkers needs to be obtained in biological samples that can be reasonably obtained, which is difficult when looking at brain tissue. We address these limitations by analyzing a wide variety of data sets to meet these conditions with data sets composed of medicated and non-medicated patients, several different biological samples, and different BD subtypes.

Thus, this study analyzes key gene pathways in BD through bioinformatics approaches. Understanding what genes are significant for BD and what genes differentiate BD 1 and BD 2 could potentially lead to better diagnostic methods and novel biomarkers by providing novel insights about gene networks relevant to the pathogenesis of BD. Here, we identify these genes through differential expression analysis and analysis of protein-protein interaction networks. These techniques are applied to 17 sets of data and are compared against each other to identify common genes and genetic pathways.

## Methods

Data sets were searched in the National Center for Biotechnology Information (NCBI) for data relating to Bipolar Disorder and Cyclothymia, resulting in the discovery of 37 genetic expression data sets. Data sets were obtained from the Gene Expression Omnibus (GEO). Only 16 of these data sets contained data formatted for analysis with GEO2R. Of these data sets, only 14 contained significant results with our statistical cutoffs (p < 0.05 and LogFC > |0.5|), and only 1 contained separable BD 2 expression data (GSE39653)^33, 34^. We also included an additional data set that was not analyzed using GEO2R that met the statistical cutoffs (p < 0.05 and LogFC > |0.5|). PubMed was also searched using the same terms to find data sets not published in GEO. Searching for Cyclothymia or Cyclothymic disorder returned zero results on NCBI’s collection of GEO Accessions.

For readability, GEO accession numbers are referred to by the name of an author of the study. These studies contain a multitude of authors who are also important, but for the clarity of this study, the data sets are named after specific authors. Data sets that are listed with samples of Bipolar Disorder are due to the data set not specifying which samples it contains that are BD 1 or BD 2, or if both subtypes are contained in the data set. Some data sets also contained samples from patients with SZ or MDD which we filtered from analysis. All patients in these GEO data sets were clinically diagnosed; most under the criteria of the DSM-IV (Supplementary Table 3).

### Abdolmaleky:

GSE120341 contains samples of prefrontal cortex from 35 patients with BD and 25 control samples^35^. None of the DEGs met our filter criteria stated in our differential expression analysis.

### Arion:

GSE87610 contains samples of dorsolateral prefrontal cortex from 17 patients with Bipolar Disorder Type 1, 2 patients with BD NOS, and 19 control samples^36, 37^.

### Bahn:

GSE5392 contains samples of the orbitofrontal cortex and dorsolateral prefrontal cortex from 40 patients with Bipolar Disorder and 42 control samples^38^.

### Bousman:

GSE18312 contains samples of peripheral blood mononuclear cells (PBMC) from 9 patients with BD and 8 control samples^39, 40^. 6 patients diagnosed with BD are reported to have had at least 1 episode of psychosis by the criteria of the DSM-IV, although the specific samples are not specified. The study contained samples collected from San Diego, USA and Taiwan, however, only samples from San Diego were published as a GEO Ascension.

### Chen:

GSE35977 contains samples of the parietal cortex from 45 patients with Bipolar Disorder and 50 control samples^41^. Zero enriched GO terms were identified in this data set.

### Chen and Liu:

GSE35978 contains samples of the cerebellum and parietal cortex from 82 patients with Bipolar Disorder and 100 control samples^41^. This is comprised of two other data sets Chen (GSE35977) and Liu (GSE35974), both of which are analyzed in this study.

### Clelland:

GSE46449 contains samples of peripheral blood leukocytes from 3 never medicated patients with Bipolar Type 1 and 25 controls^42^. This data set also contains samples from 26 medicated BD 1 patients which we excluded from our analysis in order to focus on the set of never medicated patients with BD 1.

### Cruceanu:

This data set contains samples from the anterior cortex of 13 patients with Bipolar Disorder and 13 control samples^43^. This data set was not analyzed using GEO2R and we used the reported numbers in their study.

### De Baumont:

GSE62191 contains samples from the frontal cortex of 29 patients with BD and 30 control samples^44^. None of the DEGs met our filter criteria stated in our differential expression analysis.

### Hilscher:

GSE66196 contains samples from lymphocyte and fibroblast cell lines derived from 20 patients with BD and 22 controls treated with 1 mM lithium chloride^45^. Samples from lymphocytes and fibroblasts were merged for this study.

### Iwamoto:

GSE12654 contains samples of the prefrontal cortex from 11 patients with Bipolar Disorder and 15 control samples^46^.

### Kim:

GSE74358 contains iPSCs differentiated into neuroprogenitors and then into neurons from 4 samples of Old Amish Pedigree with BD 1 and 4 control samples^47^. The data set was a time series experiment, with gene expression of samples taken at different intervals. For the purposes of this study, we treated all the times as one group of bipolar samples against all the times of the control samples, rather than having many permutations of time series compared to the control.

### Liu:

GSE35974 contains samples of the cerebellum from 37 patients with Bipolar Disorder and 50 control samples^48^. Zero enriched GO terms were identified in this data set.

### Logotheti:

GSE69486 contains samples of skin fibroblasts from 10 patients with Bipolar Disorder Type 1 and 5 control samples^49^.

### Matigian:

GSE7036 contains lymphocyte samples from 3 pairs of identical twins discordant for Bipolar Disorder Type 1 (3 samples with BD 1 and 3 samples without BD)^50^. Of the three pairs, one twin would have a clinical diagnosis of Bipolar Disorder Type 1 while their twin would not.

### Savitz:

GSE39653 was constructed to look at inflammation and neurological disease related genes in depressed patients with mood disorders^34^. This data set contains PBMC samples from 4 patients with BD 1, 4 patients with BD 2, and 24 controls. Patients in the data set did not take psychotropic medicines for 3 weeks, or 8 weeks for fluoxetine, before the blood draw for sample collection.

### Vawter:

GSE78246 contains anterior cingulate cortex samples from 9 patients with BD 1 and 11 control samples^51^.

### Witt:

GSE46416 contains blood samples from 11 patients with Bipolar Disorder Type 1 in either a manic state or euthymic state. This was compared against 10 control samples^52^. The study does not explicitly state that these BD patients had BD 1, however, the study confirms the patients had manic episodes by DSM-IV diagnosis. Thus, we assume that these samples are from BD 1 patients. For our study, our analysis combined both manic and euthymic samples into one group for comparison against the controls.

Combined this totals 306 patients with Bipolar Disorder and 366 control samples, avoiding double counting the Chen and Liu data sets (Table 2). Of this total, 61 are explicitly Bipolar Disorder Type 1, 4 are explicitly Bipolar Disorder Type 2, and 2 are explicitly Bipolar Disorder Type NOS. Additionally, this leaves 15 data sets with significant differential gene expression out of the 17 data sets. Of these 15 data sets, only 13 contained more than 10 DEGs. These are the data sets on which we focus our overall analysis.

## Differential Expression Analysis

Processing and calculation of DEGs were conducted using GEO2R, a differential gene expression analysis tool^33^. We analyzed data sets using GEO2R except for the Cruceanu data set where we used the differential gene expression reported in their study. This data set reports DEGs analyzed using HTseq^43, 53^. We analyzed the Cruceanu data set to include more data sets that identified gene expression in cortical tissue.

Using GEO2R, we often selected samples for expression analysis in different combinations than in the original study. This allowed for specifically selecting samples diagnosed with specific BD subtypes or separating BD samples from other mood disorders. For example, the study conducted by Savitz et al. merged BD 1 and BD 2 samples with MDD samples, whereas we separate the BD samples for analysis^34^. GEO2R was used with default parameters which applied a log transformation to the fold change data (LogFC) and calculated the p value. We select for DEGs with a p value < 0.05 and a LogFC > |0.5|. Genes that were ambiguously reported by GEO2R were removed. Venn diagrams were generated in R using the R package VennDiagram^54, 55^. DEGs were analyzed in three separate groups: bulk, down-regulated, and up-regulated in order to identify how gene pathways were being affected.

## Protein-Protein Interaction Network

To generate a protein-protein interaction network, we used the Search Tool for the Retrieval of Interacting Genes (STRING)^56, 57^. (https://string-db.org). This tool maps proteins to other proteins using their known and predicted interactions with other proteins. We used the default parameters in STRING in this study (Confidence score > 0.4).

Deeper analysis of the STRING network was done in Cytoscape v3.9.1^58^. Using the Cytoscape package, cytoHubba, genes in the STRING network were screened to determine the 10 hub genes^59^. This is done through the calculation of the Maximal Clique Centrality (MCC) score. MCC is the default setting in cytoHubba and was shown in package developer’s study to outperform other calculated measurements^59^. This method is best for determining proteins essential to the protein-protein interaction network. We report the hub genes of each bulk data set in Table 3.

## GO Term Analysis

The GO term analysis was conducted using the STRING website using a false discovery rate of 0.05 and a Benjamini-Hochberg adjusted p value cutoff of 0.05^60^. Analysis was conducted on the protein-protein interaction network as well as the hub gene networks. We use the three GO term categories: biological process (BP), molecular function (MF), and cellular component (CC). STRING reports GO terms scored by Strength, which is calculated as the log of observed over expected genes. Observed is the number of genes in the network associated with the GO term and expected is the expected number of genes in the network that would be associated with that GO term if the network was random. Thus, a strength value greater than 0 means the GO term occurs more frequently than by random chance.

$$Strength={\text{log}}_{10}\frac{Observed}{Expected}$$

**Figure 1.**
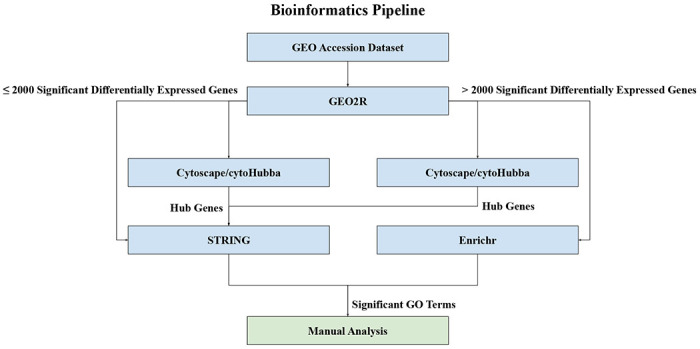
Bioinformatics Pipeline The overall bioinformatics pipeline used in this study. Data sets are analyzed using GEO2R and then enriched GO terms are identified using STRING or Enrichr depending on the number of DEGs. Hub genes are identified in the protein-protein interaction network using the cytoHubba package in Cytoscape.

However, the STRING website is limited only to generating networks of up to 2000 genes and thus any set of genes larger than 2000 was analyzed using Enrichr (https://maayanlab.cloud/Enrichr/)^61–63^. Enrichr reports an Odds Ratio instead of Strength to report the likelihood of a GO term being enriched for a set of genes. Odds Ratios for each GO term are calculated with a rank based on calculating the Fisher’s Exact Test over many random sets of genes to obtain a mean rank and standard deviation for each GO term^64^. This gives an expected likelihood of each GO term appearing for a random set of genes. The Odds Ratio for a given set of genes is then calculated as a z-score to assess the deviation of the enrichment of GO terms in that set of genes compared to the expected enrichment^61^. Thus the larger the Odds Ratio, the less likely it was a random association. We selected the GO Biological Process 2021, GO Molecular Function 2021, and GO Cellular Component 2021 libraries in Enrichr for this study. Figure 1 depicts an overview of the pipeline.

## Results

The observed DEGs led us to focus here on five gene pathways for detailed analysis: 1) Immune pathway, 2) RNA pathway, 3) Neurocognitive pathway, 4) Metal ion pathway, and 5) ATP pathway. Table 4 shows which pathways were enriched in each of the analyzed data sets. We chose the five pathways based on our data analysis of the resulting GO terms and noted frequently enriched types of GO terms. The immune pathway refers to GO terms that we identified that are related to the immune system or inflammation. The RNA pathway refers to GO terms that are related to RNA, such as its synthesis, cleavage, or structure. The neurocognitive pathway refers to the set of GO terms that we identified that are related to neurons, neurotransmitters, cognition, behavior, and other hormones related to brain function. The metal ion pathway refers to GO terms we identified as related to the biological and molecular functions that involve metal ions such as lithium and calcium. Due to lithium being one of the major and few treatments for BD, we kept track of pathways related to metal ion response or usage. Finally, the ATP pathway refers to GO terms involved in ATP synthesis, its processing, and mitochondria in general. Of the data sets that had more than 10 DEGs, 15 were examined (Table 4): 12 were enriched for immune system related terms, 12 were enriched for RNA related terms, nine were enriched for neurocognitive related terms, nine were enriched for metal ion related terms, and five were enriched for ATP related terms.

A full list of every significant DEGs in these data sets can be found in Supplementary Table 1. A full list of every significantly enriched GO term found in these data sets is presented in Supplementary Table 2. Detailed demographic information about each data set is summarized in Supplementary Table 3. The hub genes for each bulk, down-regulated, and up-regulated DEG comparison are listed in Supplementary Table 4. The protein-protein interaction network for every data set can be found in the supplementary files.

### Arion Study - Dorsolateral Prefrontal Cortex Bipolar Type 1 vs Control

**Figure 2.**
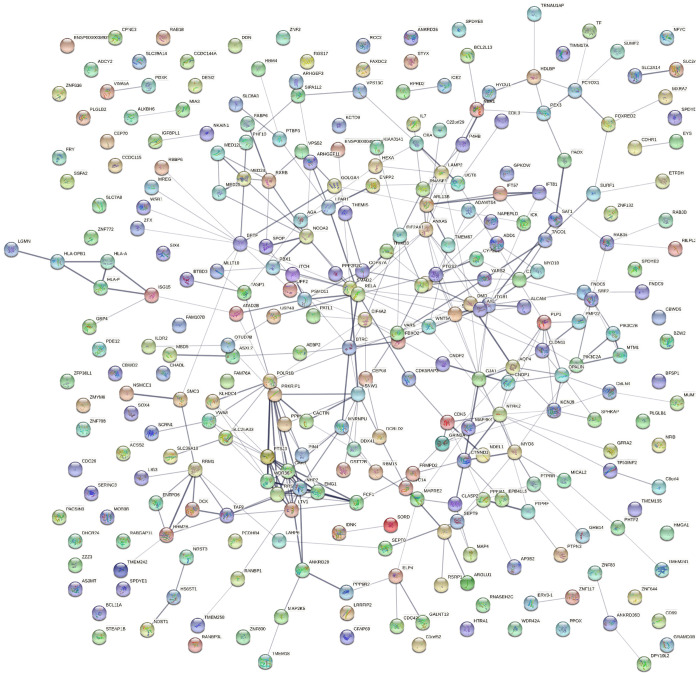
Arion Bipolar Type 1 vs Control Protein-Protein Interaction Network Whole STRING interaction network of bulk DEGs in the Arion BD 1 vs Control with LogFC > |0.5|. Confidence score > 0.4.

Overall, the Arion data set was primarily enriched for terms related to RNA, specifically ribosomal RNA (rRNA). Figure 2 demonstrates the entire protein-protein interaction network of DEGs in this comparison.

#### Bulk

The bulk set of GO terms shows that the top CC is located in the pre-ribosome (Strength = 0.77). Further analysis using the hub genes, listed in Table 3, reveals that $\frac{13}{17}$ of the BP GO terms are related to rRNA. The large majority of terms are related to the modification of rRNA through splicing, cleavage, modification, and methylation. Each of these terms have a Strength > 2. Additionally, terms related to the biogenesis of the small and large ribosomal subunit (Strength = 2.05 and Strength = 1.91, respectively), as well as the export of the subunit from the nucleus (Strength = 2.48), are also significant.

**Figure 3.**
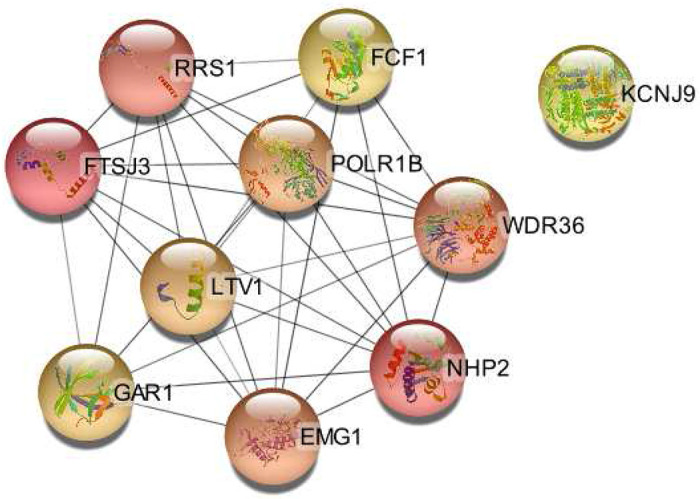
Arion Hub Genes Bipolar 1 vs Control Protein-Protein Interaction Network Whole STRING gene interaction network of top 10 key hub genes of Arion BD 1 vs Control with LogFC > |0.5|. Confidence score > 0.4.

Figure 2 shows the entire protein-protein interaction network of the bulk DEGs in this data set. From this, cytoHubba was used to generate Figure 3, which shows the 10 top key hub genes calculated using the top 10 MCC scored genes^59^.

#### Down-regulated

The down-regulated DEGs only had four enriched CC GO terms which were placed primarily in intracellular organelles, although their Strength is low between 0.07 and 0.08. In contrast, their hub genes are significantly enriched for GO terms related to rRNA modification and the ribosomal units. $\frac{14}{15}$ of the BP GO terms were related to RNA. Terms for rRNA modification were strong such as rRNA modification (Strength = 2.35) and endonucleolytic cleavage (Strength = 2.64). The same small and large ribosomal subunit terms were observed (Supplementary Table 2).

The top MF GO term was box h/aca snoRNA binding (Strength = 2.89). The strongest CC terms were identified in box h/aca complexes (Strength = 2.99), which is where the site-specific pseudo-uridylation occurs^65^. The disruption of this complex leads to the accumulation of mutant RNAs^65^.

#### Up-regulated

Zero significantly enriched GO terms were identified in up-regulated DEGs of the Arion data set in the full list of up regulated DEGs or their hub genes.

### Arion Study - Dorsolateral Prefrontal Cortex Bipolar Type NOS vs Control

This data set contains only 2 samples from patients with BD NOS. However, we investigate this low statistical power comparison due to the lack of other BD NOS samples.

#### Bulk

The large majority of GO terms for both the whole set of DEGs and the hub genes are related to RNA splicing, similar to the Arion BD 1 vs Control comparison (Supplementary Table 2).

The strongest BP GO term is the regulation of RNA splicing (Strength = 0.42), followed by splicing via transesterification reactions (Strength = 0.32) and mRNA splicing via splicosome (Strength = 0.32) following closely after. The terms for mRNA processing (Strength = 0.31), RNA processing (Strength = 0.27), mRNA metabolic process (Strength = 0.25), and RNA metabolic process (Strength = 0.21) are also seen. The strongest CC is the cytoplasmic stress granule (Strength = 0.49), which are ribonucleic protein granules that form as a response to stress to assist in regulating RNA homeostasis^66^.

The enriched cellular components enriched are in several types of synapses. Specifically, the asymmetric synapse (Strength = 0.29), neuron to neuron synapse (Strength = 0.27), and post-synapse (Strength = 0.2) are enriched cellular components.

Their hub genes are similarly related to RNA, with $\frac{14}{16}$ GO terms related to RNA. Processing of small subunit (ssu)-rRNA, 5.8s rRNA, and large subunit (lsu)-rRNA, make up the majority of biological processes. The top BP GO term is the endonucleolytic cleavage to generate mature 5-end of ssu-rRNA from (ssu-rRNA, 5.8s rRNA, lsu-rRNA) (Strength = 2.99). This is followed by the maturation of 5.8s rRNA (Strength = 2.24) and rRNA processing (Strength = 1.81). Many cellular components refer to the pre-ribosome, such as the small subunit precursor (Strength = 2.45) (Supplementary Table 2).

#### Down-regulated

In the down-regulated DEGs, the terms are primarily related to mRNA (Supplementary Table 2). These terms include mRNA splicing via the spliceosome (Strength = 0.55), mRNA processing (Strength = 0.51), and mRNA metabolic process (Strength = 0.42). The top CC is the spliceosomal complex (Strength = 0.58).

Interestingly, the top MF is ATPase binding (Strength = 0.73), which is followed by mRNA binding (Strength = 0.45) and then RNA binding (Strength = 0.31). ATPase is a class of enzymes that binds to ATP and converts it to ADP, releasing energy in the process. We decided to monitor terms related to ATP and the mitochondria since neurons are very energy intensive cells. The observation that ATPase binding is down-regulated in this neuronal data set implies that neurons are less able to utilize energy.

In the set of down-regulated hub genes, $\frac{12}{15}$ terms are related to RNA, rRNA, and ncRNA. These terms refer to the maturation and processing of rRNA (Supplementary Table 2). For example, the top BP is the maturation of lsu-rRNA from tri-cistronic rRNA transcript (ssu-rRNA, 5.8s rRNA, lsu-rRNA) (Strength = 2.42) and the top MF is RNA helicase activity (Strength = 1.9).

#### Up-regulated

In the up-regulated DEGs, many terms are related to metabolic processes of molecules in the cell. The top BP GO term is the cellular macromolecule biosynthesis process (Strength = 0.22). This is followed by several other cellular biosynthesis processes, such as for organic substances. Additionally, several metabolic process terms are enriched in the biosynthesis processes. (Supplementray Table 2) The strongest of these is the RNA metabolic process (Strength = 0.24).

This set’s hub genes were related to rRNA cleavage, specifically in ssu-rRNA, 5.7 rRNA, and lsu-rRNA. These make up $\frac{8}{10}$ of the significant GO terms in these hub genes (Supplementary Table 2).

### 0.1 Bahn Study - Dorsolateral Prefrontal Cortex and Orbitolateral Cortex Bipolar vs Control

This data set has previously been analyzed using GEO2R and STRING by several other studies^29, 30^. The majority of GO terms we identified were related to the neurocognitive pathway.

#### Bulk

The majority of BP terms are related to the secretion of neurotransmitters, the secretion of hormones, and human behavior (Supplementary Table 2). The strongest enriched BP is the positive regulation of corticosterone secretion (Strength = 2.44). Corticosterone is a steroid produced in the adrenal glands that is released in a stress response. It is known to have effects on memory, affecting memory recognition and consolidation. Additionally, the BP GO terms adult behavior (Strength = 1.18) and locomotory behavior (Strength = 1.03) are enriched. Glutamate secretion (Strength = 1.59), chemical synaptic transmission (Strength = 1.08), and positive regulation of synaptic transmission (Strength = 1.15) are also enriched. The strongest MF GO term is inhibitory extracellular ligand-gated ion channel activity (Strength = 1.92). The top CC term is the glycinergic synapse (Strength = 2.08), followed by the GABA-ergic synapse (Strength = 1.35), and synaptic membrane (Strength = 0.93).

**Figure 4.**
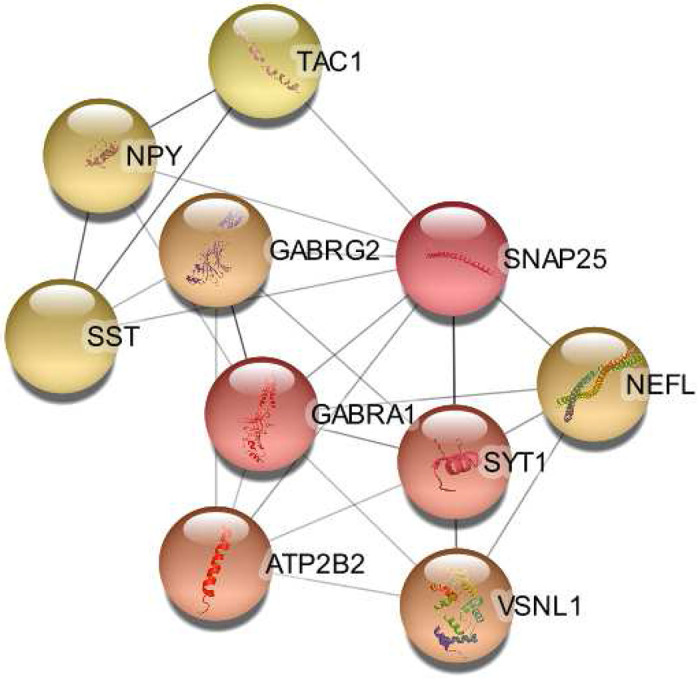
Bahn Hub Genes Bipolar vs Control Protein-Protein Interaction Network Whole STRING gene interaction network of top 10 key hub genes of Bahn BD vs Control with LogFC > |0.5|. Confidence score > 0.4.

The hub genes primarily identify terms related to synaptic transmission. Enriched are the inhibitory synapse assembly (Strength = 2.51), synaptic transmission, GABA-ergic (Strength = 2.45), positive regulation of synaptic transmission (Strength = 1.6), chemical synaptic transmission (Strength = 1.52), nervous system process (Strength = 0.94), and nervous system development (Strength = 0.76). The top CC is the GABA-A receptor complex (Strength = 2.48), followed by the dendrite membrane (Strength = 1.95) and GABA-ergic synapse (Strength = 1.9). More components of the neuron are identified, with synaptic membrane (Strength = 1.4), postsynaptic membrane (Strength = 1.32), axon (Strength = 1.18), post-synapse (Strength = 1.18), pre-synapse (Strength = 1.12), and neuron projection (Strength = 0.93) being enriched. Figure 4 shows the interaction network of the 10 hub genes that were identified in this bulk data set.

#### Down-regulated

The set of down-regulated DEGs shows similar GO term results as in the bulk set of DEGs. The strongest enriched BP is the positive regulation of corticosterone secretion (Strength = 2.46). Additionally, the BP GO terms adult behavior (Strength = 1.2), locomotor behavior (Strength = 1.05), and behavior (Strength = 0.84) are enriched. Glutamate secretion (Strength = 1.61), chemical synaptic transmission (Strength = 1.1), and positive regulation of synaptic transmission (Strength =1.17) are also enriched. The strongest MF GO term is inhibitory extracellular ligand-gated ion channel activity (Strength = 1.84). The top CC term is the glycinergic synapse (Strength = 2.09). Additionally, other related terms are found for the GABA-A receptor complex (Strength = 1.83) and GABA-ergic synapse (Strength = 1.37).

The hub genes are identical to the hub genes of the bulk set of DEGs. This is because the Bahn data set only contains 50 DEGs, of which 48 DEGs are down-regulated. This resulted in the same hub genes for both sets and explains why the GO terms are so similar.

#### Up-regulated

Zero significantly enriched GO terms were identified in up-regulated DEGs of the Bahn data set in the full list of up-regulated DEGs or their hub genes. The only two up-regulated DEGs are MT1X and ETNPPL.

### 0.2 Bousman Study - PBMC Bipolar vs Control

#### Bulk

The strongest BP is DNA replication-dependent nucleosome assembly (Strength = 1.67). The second strongest is rDNA heterochromatin assembly (Strength = 1.61). None of the MF GO terms are significant, and the only significant CC is the nuclear nucleosome (Strength = 1.6) (Supplementary Table 2).

The hub genes show terms related to the immune system and RNA (Supplementary Table 2). The terms interleukin-7 mediated signaling pathway (Strength = 2.12) and regulation of immune system process (Strength = 1.01) are enriched. The RNA related BP is the regulation of gene silencing by miRNA (Strength = 1.93). The top MF is IgE binding (Strength = 2.89) which refers to the binding function of antibody proteins. The binding to IgE antibodies in mast cells cause the secretion of type 2 cytokines. The top CC is the nuclear nucleosome (Strength = 2.41).

#### Down-regulated

In the set of down-regulated DEGs, there are only three GO terms, all BPs. These are the retina homeostasis (Strength = 1.48), detection of chemical stimulus involved in sensory perception (Strength = 0.91), and detection of stimulus (Strength = 0.78).

The hub genes of down-regulated DEGs have one term, which is the MF GO term IgE binding (Strength = 2.89).

#### Up-regulated

Terms related to RNA and the immune system are enriched in both the full set of up-regulated DEGs and their hub genes. Negative regulation of hemopoiesis (Strength = 1.05) and the regulation of immune system processes (Strength = 0.69), were enriched BP terms in the full set of up-regulated DEGs. In addition, the BP term regulation of gene silencing by miRNA (Strength = 1.61) and the CC cytosolic ribosome (Strength = 1.46) were enriched. While hemopoiesis is not strictly related to the production of white blood cells as it could be the up-regulation of red blood cells instead, it is worth considering since the regulation of immune system processes is also enriched.

The hub genes are enriched for the BP terms interleukin-7 mediated signaling pathway (Strength = 2.12) and the regulation of the immune system process (Strength = 0.96). The RNA related terms saw enrichment of the BP regulation of gene silencing by miRNA (Strength = 1.93), and the cellular components polysomal ribosome (Strength = 2.09) and cytosolic large ribosomal subunit (Strength = 1.84).

### 0.3 Chen and Liu Parietal Cortex and Cerebellum Bipolar vs Control

Two significant DEGs were identified and both were down-regulated. These were the genes HLA-DRA and HLA-DQA1 (Table 3). These two genes are part of the Major Histocompatibility Complex, Class II, which initiates an immune response by interaction with T cells in order for T cells to recognize antigens. They are found in antigen-processing cells and can also be induced by interferon-*γ*. Both of these genes were down regulated with LogFC = −0.52065153. HLA-DRA was previously reported to be down-regulated in BD patients compared to controls in samples taken from the dorsolateral prefrontal cortex^67^.

### 0.4 Clelland Study - Blood Never Medicated Bipolar Type 1 vs Control

#### Bulk

**Figure 5.**
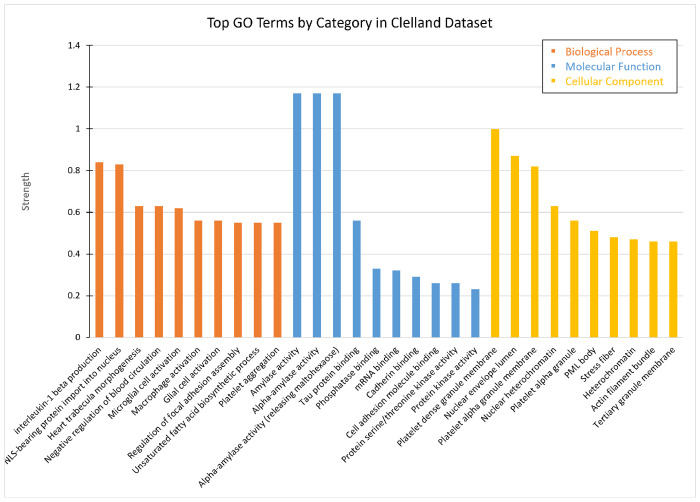
Top 30 Clelland Never Medicated BD 1 vs Control Bulk GO Terms The top 10 BP, MF, and CC GO terms for Clelland Never Medicated BD 1 vs Control Bulk GO terms.

A number of terms are related to the immune cell activation and production of interleukins, but also terms related to mRNA splicing, are enriched. $\frac{60}{339}$ of the BP GO terms were related to the immune system, while 15 of these BP terms were related to RNA. The top 10 BP, MF, and CC terms are shown in Figure 5.

The strongest BP GO term is the term interleukin-1*β* production (Strength = 0.84). Following this, other enriched terms are microglial cell activation (Strength = 0.62), macrophage activation (Strength = 0.56), and glial cell activation (Strength = 0.56). Many other terms refer to the activation of other immune cells and cytokine response. Of the RNA terms, the strongest is the regulation of alternative mRNA splicing via splicosome (Strength = 0.52), followed by the regulation of mRNA splicing via spliceosome (Strength = 0.48), regulation of mRNA processing (Strength = 0.43), and regulation of RNA splicing (Strength = 0.41). Figure 5 shows the top 10 BP, MF, and CC GO terms in this bulk data set.

The hub genes, as seen in Table 3, contain $\frac{25}{57}$ terms related to RNA pathways. The top biological processes are the negative regulation of mRNA splicing via spliceosome (Strength = 2.41), 3-UTR-mediated mRNA stabilization (Strength = 2.36), and alternative mRNA splicing via splicosome (Strength = 2.34). The top MF terms are the N6-methyladenosine-containing RNA binding (Strength = 2.69), pre-mRNA intronic binding (Strength = 2.55), and pre-mRNA binding (Strength = 2.33). Additionally, the CC terms catalytic step 2 splicosome (Strength = 1.95) and ribonucleoprotein complex (Strength = 1.24) are enriched. We note that many of the RNA terms have enriched cellular components in the nucleus, which may indicate where the effects of these DEGs may be occurring (Supplementary Table 2).

#### Down-regulated

In the set of down-regulated DEGs, terms related to the immune system, RNA, metal ions, and ATP were found. The top BP is protein retention in the Golgi apparatus (Strength = 1.19). The BP terms regulation of alternative mRNA splicing via splicosome (Strength = 0.6), regulation of mRNA processing (Strength = 0.51), and regulation of RNA splicing (Strength = 0.46) are also identified. In addition, the immune related biological GO terms regulation of myeloid cell differentiation (Strength = 0.36), hemopoiesis (Strength = 0.23), and immune system development (Strength = 0.21) also appear. Phosphorus metabolic process (Strength = 0.15), a metal ion related term, is one of the BP terms. The top MF term is alpha-amylase activity (Strength = 1.29). The MF terms mRNA binding (Strength = 0.34), transferase activity transferring phosphorus-containing groups (Strength = 0.19), and ATP binding (Strength = 0.16) are also found. The top CC is the platelet-dense granule membrane (Strength = 1.11).

Within the hub genes, $\frac{23}{37}$ GO terms were related to mRNA or RNA, primarily splicing and transport (Supplementary Table 2). The top BP is alternative mRNA splicing via splicosome (Strength = 2.34), which is followed by negative regulation of mRNA splicing via splicosome (Strength = 2.23), and mRNA splice site selection (Strength = 2.06). The top MF is the G-rich strand telomeric DNA binding (Strength = 2.59), which is followed by pre-mRNA intronic binding (Strength = 2.55) and miRNA binding (Strength = 2.1). Some CC are the catalytic step 2 splicosome (Strength = 1.83), spliceosomal complex (Strength = 1.61) and ribonucleoprotein complex (Strength = 1.16).

#### Up-regulated

These up-regulated GO terms contain many immune related terms, but also contain many CC terms connected to the synapse (Supplementary Table 2). The top BP GO term is interleukin-1 beta production (Strength = 1.35). This is followed by the positive regulation of type 2 immune response (Strength = 1.22), positive regulation of interferon-beta production (Strength = 0.99), and regulation of cytokine production involved in inflammatory response (Strength = 0.94). Additionally, the positive regulation of interleukin-1 beta (Strength = 0.86) and interleukin-6 (Strength = 0.81) were identified. The top MF term is cell adhesion molecule binding (Strength = 0.44). The top CC is the inflammasome complex (Strength = 1.25). In addition, the CC terms postsynaptic endosome (Strength = 1.13), intrinsic component of synaptic vesicle membrane (Strength = 0.84), synaptic vesicle (Strength = 0.56), distal axon (Strength = 0.45), and presynapse (Strength = 0.38) are enriched.

This supports that these immune system effects are not restricted to peripheral blood but occur in the brain as well, and more importantly it shows that these effects in the brain can be identified downstream in peripheral blood samples. This lends more evidence to the notion that the blood could contain a suitable biomarker for identifying BD.

In the hub genes, many terms were found to be related to the immune system (Supplementary Table 2). Similar to other sets of immune terms, the production of cytokines appears to be a strongly enriched process in individuals with BD. The top BP term is NLRP3 inflammasome complex assembly (Strength = 3.29). This is followed by cytokine secretion involved in immune response (Strength = 2.99), interleukin-1 beta production (Strength = 2.72), and positive regulation of type 2 immune response (Strength = 2.39). The top CC is the inflammasome complex (Strength = 2.59) followed by the phagocytic vesicle (Strength = 1.63).

### 0.5 Cruceanu Study - Anterior Cortex Bipolar vs Control

This is the only data set we analyzed not using GEO2R but instead used the reported results from the authors’ study which used HTSeq.

#### Bulk

Due to the amount of DEGs, Enrichr was used to analyze the bulk set of DEGs of this data set. $\frac{33}{89}$ terms were related to neurocognitive pathways and $\frac{15}{89}$ terms were related to metal ions (Supplementary Table 2). The top BP is the adenylate cyclase-inhibiting G protein-coupled acetylcholine receptor signaling pathway (Odds Ratio = 24.974). Following that, the BP terms related to neurocognitive pathways are the inhibitory synapse assembly (Odds Ratio = 14.573), synaptic transmission, GABAergic (Odds Ratio = 14.573), regulation of dopamine secretion (Odds Ratio = 6.552), and synaptic vesicle exocytosis (Odds Ratio = 5.569) The MF terms include G protein-coupled neurotransmitter receptor activity (Odds Ratio = 20.802), GABA-A receptor activity (Odds Ratio = 9.263), and neurotransmitter receptor activity involved in regulation of postsynaptic membrane potential (Odds Ratio = 5.124). The top CC is the clathrin-sculpted gamma-aminobutyric acid transport vesicle (Odds Ratio = 13.867), followed by the synaptic vesicle membrane (Odds Ratio = 7.225), synaptic membrane (Odds Ratio = 5.209), and dendrite membrane (Odds Ratio = 5.095). Similar to the Bahn data set, terms related to GABA, the synapse, and neurotransmitters were found to be significant in this set of DEGs.

Additionally, the metal ion BP terms include the cellular response to zinc ion (Odds Ratio = 6.663), regulation of calcium ion-dependent exocytosis (Odds Ratio = 6.027), and potassium ion transmembrane transport (Odds Ratio = 2.824). The MF terms include GABA-gated chloride ion channel activity (Odds Ratio = 13.330), potassium ion leak channel activity (Odds Ratio = 6.475), and calcium-dependent phospholipid binding (Odds Ratio = 3.243). The data also includes two RNA related cellular components which are the cytosolic small ribosomal subunit (Odds Ratio = 3.572) and small ribosomal subunit (Odds Ratio = 3.031)

The hub genes were once again analyzed using STRING. $\frac{14}{23}$ terms in the hub genes were related to ribosomal assembly and the ribosomal structure. The top BP is the ribosomal small subunit assembly (Strength = 2.34), followed by nuclear-transcribed mRNA catabolic process, nonsense-mediated decay (Strength = 2.22), ribosomal large subunit assembly (Strength = 2.15), and ribonucleoprotein complex assembly (Strength = 1.5). There are two significant MF GO terms which are the structural constituent of ribosome (Strength = 2.09) and RNA binding (Strength = 1.07). The enriched cellular components are identified in two key places: the ribosomal subunits and the synapse. The top CC is the polysomal ribosome (Strength = 2.39) and followed by the cytosolic ribosome (Strength = 2.3). Additionally, the CC terms postsynaptic density (Strength = 1.45) and synapse (Strength = 0.94) are enriched. This shows that some of these effects in the ribosome assembly are taking place inside neurons around the synapse.

#### Down-regulated

Many terms are related to synaptic signaling, neurotransmitter secretion, and metal ion functions (Supplementary Table 2). The top BP is the adenylate cyclase-inhibiting G protein-coupled acetylcholine receptor signaling pathway (Strength = 1.16). Following this, the BP terms regulation of synaptic vesicle priming (Strength = 1.14), synaptic transmission, GABAergic (Strength = 1.09), spontaneous synaptic transmission (Strength = 1.08), and inhibitory synapse assembly (Strength = 1.05) are significantly enriched in this set of down-regulated DEGs. This is also followed by the regulation of dopamine secretion (Strength = 0.93), glutamate secretion (Strength = 0.92), and neurotransmitter receptor transport to postsynaptic membrane (Strength = 0.87). There are also BP terms related to nerve development that are enriched in this down-regulated data set. Axonogenesis (Strength = 0.5), nerve development (Strength = 0.5), and dendrite development (Strength = 0.49) are significant enriched terms. These results indicate worse neuronal health in people with BD.

Additionally, one immune related BP GO term cellular response to histamine (Strength = 1.08) is identified, but it could be enriched concerning the neurotransmitter functions of histamine instead of the immune related functions.

Several terms related to metal ions were found with the strongest being calcium ion-regulated exocytosis of neurotransmitters (Strength = 1.02). Several terms related to the regulation of calcium ion concentration and calcium ion-regulated exocytosis are also found (Supplementary Table 2). Additionally, terms related to other metal ions such as potassium, calcium, and sodium are found in this set of down-regulated DEGs (Supplementary Table 2). Several behavior terms are also found in this down-regulated DEG set as well, such as long-term memory (Strength = 0.75), learning (Strength = 0.68), and locomotory behavior (Strength = 0.63).

Many down-regulated BP terms are related to synaptic transmission, with strong terms related to GABA and dopamine. Neuron generation is also key in these GO terms. Additionally, neurotransmitter secretion and production are enriched in this set of down-regulated DEGs. This is consistent with the wider literature about the importance of the GABAergic pathway in BD^68–71^. Additionally, several metal ion terms were enriched as well. Calcium ion dependent processes, such as the calcium ion regulated exocytosis of neurotransmitters, are more frequently enriched than other metal ions.

In the hub genes, $\frac{48}{89}$ GO terms are related to neurocognitive pathways. The top BP term is pre-synaptic dense core vesicle exocytosis (Strength = 2.89). This is followed by synaptic vesicle maturation (Strength = 2.85), calcium ion regulated exocytosis of neurotransmitters (Strength = 2.59), and synaptic vesicle priming (Strength = 2.56). There are additional terms related to neurotransmitter secretion, such as glutamate secretion (Strength = 2.39), positive regulation of neurotransmitter secretion (Strength = 2.39), and regulation of dopamine secretion (Strength = 2.04).

In the MF GO terms, the top term is syntaxin-1 binding (Strength = 2.58) which is followed by calcium-dependent protein binding (Strength = 1.97). Syntaxin is a protein involved in the release of neurotransmitters, so the down-regulation of this function could lead to reduced neuronal signaling. The top cellular components refer to the vesicles, each with a Strength > 2 (Supplementary Table 2). Other terms are related to the synapse as a whole and neurons (Supplementary Table 2).

The clear pattern in these hub genes is the down-regulated role of synaptic vesicles, which in the broader picture is the down-regulated synaptic signaling. Consistently, neurotransmitter secretion is down-regulated and the ability to release them is hindered, which implicates that BD is associated with reduced neuron-to-neuron communication.

#### Up-regulated

In contrast to the down-regulated set of DEGs in this data set, there is one term related to neurocognitive pathways in this up-regulated set of enriched GO terms. This is the BP retinal bipolar neuron differentiation (Strength = 1.54) which refers to the neurons in the eyes. Instead, terms are enriched for the other four pathways of interest in this study. This supports the conclusions found in the down-regulated set of DEGs in this data set as those terms are uniquely down-regulated.

The top immune BP is T cell extravasation (Strength = 1.34), followed by positive regulation of T cell cytokine production (Strength = 1.06), positive regulation of cytokine production involved in immune response (Strength = 0.84), and positive regulation of interleukin-1 beta production (Strength = 0.8). There are further terms related to the cellular response to different cytokines such as interferon-*γ*, neutrophils, and leukocytes (Supplementary Table 2). The MF term immune receptor activity (Strength = 0.68) is also enriched. As in the prior data sets, immune responses seem to be up-regulated in people with BD which is consistent with prior literature^18, 21^.

Several BP terms related to RNA are identified. These are the nuclear-transcribed mRNA catabolic process, nonsense-mediated decay (Strength = 1.07), nuclear-transcribed mRNA catabolic process (Strength = 0.89), mRNA catabolic process (Strength = 0.86), and mRNA metabolic process (Strength = 0.41). The top MF is the structural constituent of ribosome (Strength = 0.92). The top four CC terms are located in the ribosome, which are the cytosolic small ribosomal subunit (Strength = 1.25), polysomal ribosome (Strength = 1.21), cytosolic ribosome (Strength = 1.14), and cytosolic large ribosomal subunit (Strength = 1.06). This follows a similar pattern as identified in the prior data sets where the ribosome is a critical CC and many of the involved processes affect mRNA. What differentiates this data from those of Arion and Clelland is that these terms are appearing in the up-regulated set of DEGs. It could possibly be explained as the top BP term is related to the decay of mRNA, which could cause disruption if that process is up-regulated.

Metal ion terms were strongly enriched, such as cellular response to zinc, copper, and cadmium ions (Supplementary Table 2). For example, the top BP term of metal ions is the detoxification of copper ions (Strength = 1.46). Rahnama et al. analysis of the Bahn data set also identified that the main enriched BP in the up-regulated DEGs was the cellular response to metal ions such as copper, zinc, calcium, magnesium, and manganese^29^. Their results indicated that most of the metal ion response terms were up-regulated, which we see in this Cruceanu data set, except for calcium response which was down-regulated.

Several terms related to mitochondria are also identified. The strongest BP term of these is the mitochondrial electron transport, NADH to ubiquinone (Strength = 0.85), and then mitochondrial ATP synthesis coupled electron transport (Strength = 0.82). In the cellular components, the terms mitochondrial respiratory chain complex I (Strength = 0.86), mitochondrial respirasome (Strength = 0.74), and inner mitochondrial membrane protein complex (Strength = 0.69) are enriched.

Among the hub genes, $\frac{10}{16}$ are related to RNA. The top BP is the ribosomal small subunit assembly (Strength = 2.34) which is followed by nuclear-transcribed mRNA catabolic process and nonsense-mediated decay (Strength = 2.22). The two MF terms are the structural constituent of ribosome (Strength = 2.09) and RNA binding (Strength = 1.03). The CC terms related to RNA are the top terms of the CC terms. They are the polysomal ribosome (Strength = 2.56), cytosolic small ribosomal subunit (Strength = 2.4), cytosolic large ribosomal subunit (Strength = 2.24), and ribosomal subunit (Strength = 2.04).

### 0.6 Hilscher Study - Lymphocyte Fibroblast Lithium Treated Bipolar vs Control

#### Bulk

The strongest and only term for this data set is the MF term MHC class I protein complex binding (Strength = 2.64), which is immune system related. In the hub genes, the only GO term is the MF GO term MHC class I protein complex binding (Strength = 2.81). Major histocompatibility complex (MHC) I molecules display peptide fragments of proteins contained inside a cell so that cytotoxic T cells can see them. These act like windows into the cell essentially. This allows T cells to see what cells need to be destroyed, such as in a viral infection.

#### Down-regulated

Zero significant enriched GO terms for down-regulated DEGs or their hub genes were found.

#### Up-regulated

In both the up-regulated DEGs and the hub genes of it, there was only one enriched term. This was again the MF term MHC class I protein complex binding (Strength = 6).

Thus, this section of the immune system was found up-regulated in this sample of lymphocytes and fibroblasts. Interestingly, we saw that the MHC class II proteins were also enriched in the Chen and Liu data set. However, these proteins were instead down-regulated in that data set.

### 0.7 Iwamoto Study - Prefrontal Cortex Bipolar vs Control

As in the Cruceanu data set, the five pathways of interest are enriched in this data set.

#### Bulk

GO terms are enriched in the immune, RNA, neurocognitive, and metal ion pathways in this set of data. The strongest biological processes GO terms are the negative regulation of glycogen metabolic process (Strength = 1.35) and the regulation of complement-dependent cytotoxicity (Strength = 1.35). Complement-dependent cytotoxicity is a function of antibodies once they bind to a target antigen to kill the target cell. Additionally, the BP terms natural killer cell activation involved in immune response (Strength = 1), regulation of interferon-gamma-mediated signaling pathway (Strength = 0.97), and T cell activation involved in immune response (Strength = 0.81) are also identified. Further immune system terms are related primarily to leukocytes, cytokines, and T cells (Supplementary Table 2). The MF GO term cytokine receptor binding (Strength = 0.53) is also identified.

Several significant RNA related GO terms were found such as the negative regulation of RNA metabolic processes (Strength = 0.32) and regulation of transcription by RNA polymerase II (Strength = 0.19). Several terms related to neurocognitive pathways are also identified. The BP GO terms nerve growth factor signaling pathway (Strength = 1.18), regulation of behavior (Strength = 0.71), negative regulation of neuron apoptotic process (Strength = 0.65), negative regulation of neuron death (Strength = 0.53), and modulation of chemical synaptic transmission (Strength = 0.39) are significantly enriched in addition to several other related terms (Supplementary Table 2).

Several metal ion related terms are found and many are related to calcium-mediated signaling, similar to the Cruceanu data set. These include the BP GO terms calcium-mediated signaling using intracellular calcium source (Strength = 1) and positive regulation of calcium-mediated signaling (Strength = 0.83) in addition to other terms related to the regulation of transport, homeostasis, and concentration of calcium ions (Supplementary Table 2).

The hub genes are primarily related to the immune system, but also include terms related to ATP and neurocognitive pathways. The strongest BP GO term is the positive regulation of interleukin-23 production (Strength = 2.89),followed by the ATP or energy-related term positive regulation of cellular respiration (Strength = 2.64). These terms are followed primarily by immune system related terms such as the positive regulation of MHC class II biosynthetic process (Strength = 2.55), negative regulation of acute inflammatory response (Strength = 2.48), macrophage differentiation (Strength = 2.35), and T cell lineage commitment (Strength = 2.27). These are followed by terms related to cytokine secretion and activation of microglial cells, macrophages, B cells, lymphocytes, and T cells which each have a Strength > 1 (Supplementary Table 2). There is an additional ATP related BP term called the positive regulation of ATP metabolic process (Strength = 2.1). Additionally, two BP terms related to neurocognitive pathways are seen which are the regulation of behavior (Strength = 1.74) and positive regulation of neuron death (Strength = 1.6). Due to many of the hub genes GO terms being strongly related to immune system pathways, it reflects the results seen in above discussed data sets.

**Figure 6.**
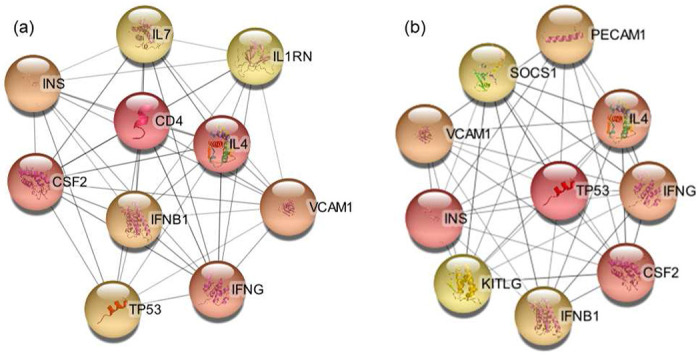
**A)** Whole STRING gene interaction network of the Iwamoto bulk data set’s top 10 hub genes with LogFC > |0.5|. Confidence score > 0.4. **B)** The protein-protein interaction network of the Iwamoto up-regulated DEGs data set’s top 10 hub genes with LogFC > |0.5|. Confidence score > 0.4.

The hub genes in Table 3 and Figure 6 contain several genes that are directly related to cytokines. IFN-*γ* is the gene that encodes the protein of the same name and is also a significant DEG in the Clelland and the Witt data sets. IL1RN, also known as IL1RA, is a competitive inhibitor of IL-1*α* and IL-1*β* which causes it to have an anti-inflammatory function. In this data set, IL1RN is down regulated which implies that the pro-inflammatory cytokine IL-1*β* will be less inhibited. IL7 functions in T cell and B cell development while also serving an anti-inflammatory role. IL7 is down regulated in this data set, which implies a pro-inflammatory profile.

Interestingly, BD is highly comorbid with Diabetes mellitus and is double to triple the prevalence compared to the general population^72–74^. Additionally, the prognosis for BD is more severe if the patient has Type 2 Diabetes^72^. INS is up regulated by LogFC = 0.90151245, meaning that the prefrontal cortex samples from this study had elevated levels of insulin. It is also a significant differentially expressed gene in the Matigian and Logotheti data sets. Of all hub genes identified across each data set, INS was the most commonly identified one (Table 5). This provides additional evidence for a mechanistic link between BD and diabetes reflected in the high rates of comorbidity.

#### Down-regulated

A similar mixture of enriched pathways was seen in the down-regulated DEGs and their hub genes as in the bulk DEG comparison. Immune system related BP terms were identified, such as leukocyte activation involved in immune response (Strength = 0.53), immune effector process (Strength = 0.41), and defense response (Strength = 0.37). The top 3 BP terms are related to neurocognitive pathways and are the nerve growth factor signaling pathway (Strength = 1.52), neurotrophin signaling pathway (Strength = 1.28), and peripheral nervous system development (Strength = 1.02). Two calcium metal ion terms are identified in the BP terms which are the regulation of calcium-mediated signaling (Strength = 0.97) and regulation of calcium ion transport (Strength = 0.67).

The hub genes have many terms related to the immune process. The strongest immune system related term in the BP terms is T cell selection (Strength = 2.17), followed by regulation of B cell activation (Strength = 1.66), regulation of lymphocyte proliferation (Strength = 1.55), and regulation of T cell proliferation (Strength = 1.55). The second strongest MF term is immunoglobulin binding (Strength = 2.33). The top two CC terms are the immunoglobulin complex (Strength = 2.55) and T cell receptor complex (Strength = 2.48).

Additionally, terms related to nerves are seen. The top BP is the nerve growth factor signaling pathway (Strength = 2.51) followed by the neurotrophin TRK receptor signaling pathway (Strength = 2.29). The top MF term is nerve growth factor receptor binding (Strength = 2.89). The third strongest CC is the synaptic vesicle (Strength = 1.46). The regulation of calcium-mediated signaling (Strength = 1.77) is also found in the BP terms.

In this down-regulated set of DEGs, nerve growth and nerve signaling-related terms are found. This implies a reduction of nerve growth and signaling in BD patients, which is consistent with the observations in the Bahn and Cruceanu data sets. Additionally, many of the immune cell activation and differentiation terms were found in this down regulated DEG set, which implies abnormality in the immune response. Specifically, it seems that immunoglobulin was affected most strongly in this down regulated DEG set.

#### Up-regulated

The immune pathway is also commonly enriched (Supplementary Table 2). We observe terms related to cytokine production and response in this up-regulated DEG set. The top BP is the negative regulation of complement-dependent cytotoxicity (Strength = 1.87), followed by T cell proliferation involved in immune response (Strength = 1.74). Additionally, the BP terms such as regulation of interferon-gamma-mediated signaling pathway (Strength = 1.23), positive regulation of lymphocyte differentiation (Strength = 0.81), B cell differentiation (Strength = 0.78), and positive regulation of cytokine production (Strength = 0.46) are observed.

RNA related terms are observed in the BP terms. mRNA transcription (Strength = 1.22), negative regulation of transcription by RNA polymerase II (Strength = 0.43), and negative regulation of RNA metabolic process (Strength = 0.42) are found in this up-regulated set. mRNA and RNA related processes have been observed before in the Arion and Clelland data sets. In those, RNA related terms were being down-regulated and in the Iwamoto data set, the negative regulation is being up-regulated. This continues the observed pattern from the above discussed data sets.

Terms related to neurocognitive pathways are observed, with the strongest biological processes of these terms being the neurophilin signaling pathway (Strength = 1.74) and positive regulation of dendritic spine maintenance (Strength = 1.74). Additionally, the regulation of behavior (Strength = 0.89), regulation of neuron apoptotic process (Strength = 0.62), and regulation of neuron death (Strength = 0.54) are in this set of BP GO terms for up-regulated DEGs. It is interesting to note that terms related to neuronal death are found in the up-regulated set of DEGs. The ATP BP term positive regulation of ATP metabolic process (Strength = 0.97) is observed as well.

The hub genes primarily consist of terms related to the immune system and consist of the genes in Figure 6. The strongest BP is the positive regulation of interleukin-23 production (Strength = 2.89) which is followed by the ATP related term positive regulation of cellular respiration (Strength = 2.64). Many of the BP terms are similar in strength and function as the bulk set of DEGs in this data set. Many terms are related to cytokine secretion and immune cell activation and differentiation in T cells, B cells, microglia, macrophages, and leukocytes. The top two molecular functions are cytokine activity (Strength = 1.62) and cytokine receptor binding activity (Strength = 1.57). The only CC is extracellular space (Strength = 0.69).

The enrichment of cytokine production terms in the up-regulated DEGs is consistent with the observations in the Clelland and Cruceanu data sets.

### 0.8 Kim Study - Neurons Time Independent Bipolar Type 1 vs Control

#### Bulk

**Figure 7.**
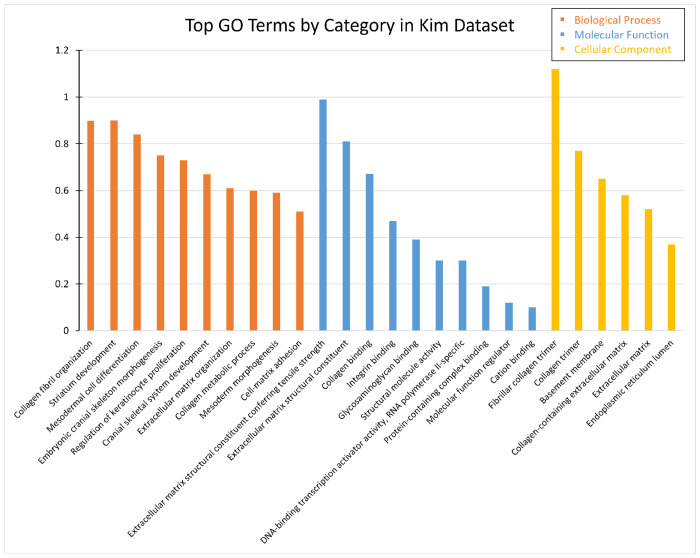
Top 30 Kim Neurons Time Independent BD 1 vs Control Bulk GO Terms The top 10 BP, MF, and CC GO terms for Kim Neurons Time Independent BD 1 vs Control Bulk GO terms.

Most GO terms in this set of DEGs are not related to the pathways of interest, but some RNA and metal ion terms are found. For example, the strongest BP term is collagen fibril organization (Strength = 0.9) and the strongest MF term is the extracellular matrix structural constituent conferring tensile strength (Strength = 0.99). The entire list of terms can be found in Supplementary Table 2. Among the pathways of interest, the BP term positive regulation of transcription by RNA polymerase II (Strength = 0.19) and molecular process term DNA-binding transcription activator activity, RNA polymerase II-specific (Strength = 0.3) are identified with relatively weak Strength values. The metal ion MF terms are cation binding (Strength = 0.1) and metal ion binding (Strength = 0.1). Most terms are instead related to collagen and skeletal functions and structure, with terms related to other cell tissue. The top 10 of each category of GO terms for this data set is shown in Figure 7.

The same pattern was observed in the hub genes in which all of the hub genes are collagen related and none of the GO terms are related to the pathways of interest.

#### Down-regulated

A similar result is seen in the down-regulated set of DEGs where many terms are related to collagen, bone, and other tissue. The only pathway of interest found in this set is the RNA related pathway. This includes the BP terms positive regulation of transcription by RNA polymerase II (Strength = 0.33) and regulation of transcription by RNA polymerase II (Strength = 0.24). It also includes the molecular functions DNA-binding transcription activator activity, RNA polymerase II-specific (Strength = 0.48), RNA polymerase II transcription regulatory region sequence-specific DNA binding (Strength = 0.39), RNA polymerase II cis-regulatory region sequence-specific DNA binding (Strength = 0.34), and DNA-binding transcription factor activity, RNA polymerase II-specific (Strength = 0.3). Similar terms have also been identified in down-regulated DEGs in the other data sets.

The hub genes in the down-regulated DEGs were identical to those in the bulk data set so they produced the same significantly enriched GO terms.

#### Up-regulated

Zero significantly enriched GO terms were observed in the set of up-regulated DEGs, but the hub genes found several significantly enriched GO terms related to neurocognitive pathways. This includes the dendritic spine organization (Strength = 2.12), neuron projection morphogenesis (Strength = 1.3), cell morphogenesis involved in neuron differentiation (Strength = 1.25), regulation of neuron project development (Strength = 1.19), and generation of neurons (Strength = 0.88). The top CC is the myelin sheath (Strength = 1.93) and is followed by the dendritic spine (Strength = 1.66), postsynaptic membrane (Strength = 1.32), and synaptic membrane (Strength =1.31).

This shows contrasting results to the Iwamoto data set where the health and generation of neurons were in the down-regulated DEG set. This is consistent with the synapse appearing as a common CC in these data sets. However, the Kim data set seems primarily to be composed of terms related to collagen and other tissues despite being taken from iPSC-derived neurons.

### 0.9 Logotheti Study - Skin Fibroblasts Bipolar Type 1 vs Control

#### Bulk

For the whole set of DEGs, no significantly enriched GO terms were found, but significantly enriched terms were found in its hub genes. The top BP term is the negative regulation of acute inflammatory response (Strength = 2.48). Additionally, metal ion related terms, especially calcium, are found in this data. The regulation of calcium ion transmembrane transport (Strength = 1.58) and regulation of ion transmembrane transport (Strength = 1.31) were observed in the BP terms. The strongest MF term is voltage-gated calcium channel activity (Strength = 2.25), followed by calcium channel activity (Strength = 1.93) and voltage-gated ion channel activity (Strength = 1.7). The top CC is the CatSper complex (Strength = 2.77), immediately followed by voltage-gated calcium channel complex (Strength = 2.58) and L-type voltage-gated calcium channel complex (Strength = 2.55). Very clearly, calcium ions are important across these BD data sets as enriched calcium ion GO terms were observed in the Cruceanu and Iwamoto data sets.

#### Down-regulated

In the set of down-regulated DEGs, only 1 term is enriched which is the CC voltage-gated calcium channel complex (Strength = 1.01). However, there are more enriched terms found in the set of down-regulated hub genes. The strongest BP term is the negative regulation of acute inflammatory response (Strength = 2.48), which is an immune related term but it is the only one in this set of hub genes. There is also one RNA related term which is the BP term positive regulation of pri-miRNA transcription by RNA polymerase II (Strength = 1.99). Additionally, there are several terms related to metal ions. There is the BP term regulation of ion transmembrane transport (Strength = 1.22). The top MF term is voltage-gated calcium channel activity (Strength = 2.25), calcium channel activity (Strength = 1.93), and voltage-gated ion channel activity (Strength = 1.7). The top CC is the CatSper complex (Strength = 2.77), followed by L-type voltage-gated calcium channel complex (Strength = 2.55) and voltage-gated calcium channel complex (Strength = 2.52). As seen in the Cruceanu and Iwamoto data sets, calcium channel related terms are found in the down-regulated sets of DEGs in people with BD.

#### Up-regulated

No significantly enriched GO terms were found in the set of up-regulated DEGs. In the hub genes, all terms are biological processes and include the positive regulation of response to external stimulus (Strength = 1.28) and homeostatic process (Strength = 0.91).

### 0.10 Matigian Study - Twin Lymphocytes Bipolar Type 1 vs Non-Bipolar Twin

GO terms can be found in this data set related to the immune system, RNA, neurocognitive, and metal ion pathways.

#### Bulk

The top BP term is the positive regulation of stem cell differentiation (Strength = 0.77). Terms related to the immune system are found in this bulk data set such as the BP term immune system development (Strength = 0.18). The RNA BP term positive regulation of transcription by RNA polymerase II (Strength = 0.14) and the MF term DNA-binding transcription factor activity, RNA polymerase II-specific (Strength = 0.16) are also found. There are many more terms related to neurocognitive pathways (Supplementary Table 2). This includes the BP terms regulation of dopamine secretion (Strength = 0.62), neurotransmitter transport (Strength = 0.41), regulation of neurotransmitter levels (Strength = 0.36), synaptic signalling (Strength = 0.26), neuron development (Strength = 0.24), and brain development (Strength = 0.21). Related CC terms include the GABA-ergic synapse (Strength = 0.45), integral component of postsynaptic membrane (Strength = 0.39), and glutamatergic synapse (Strength = 0.29). These terms are primarily composed of neurotransmitter related terms, such as GABA and dopamine, in addition to behavior and synaptic signaling. There are also several metal ion related terms such as regulation of cytosolic calcium ion concentration (Strength = 0.25) (Supplementary Table 2). Given that these samples came from lymphocytes, seeing many terms related to neurons is notable and could suggest we can see the downstream effects of BD on the brain inside of peripheral blood.

**Figure 8.**
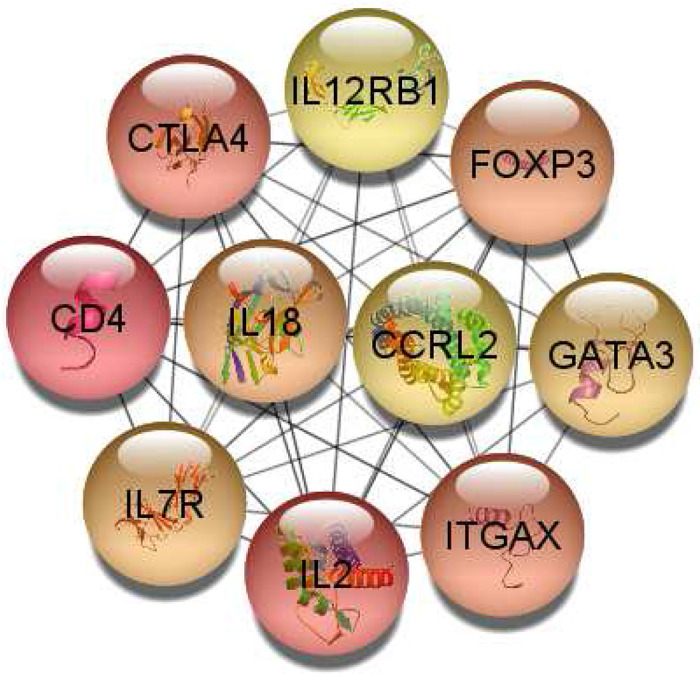
Matigian Hub Genes Bipolar Type 1 vs Control Protein-Protein Interaction Network Whole STRING gene interaction network of top 10 key hub genes with fold change > |0.5|. Confidence score > 0.4

The hub genes are primarily immune system related, making up $\frac{81}{117}$ GO terms in these hub genes. The top BP is the negative regulation of T-helper 17 cell differentiation (Strength = 2.59), followed by Type 2 immune response (Strength = 2.55), positive regulation of activated T cell proliferation (Strength = 2.39), regulation of cytokine biosynthetic process (Strength = 2.39), and many more terms related to T cells and cytokines (Supplementary Table 2). The MF terms are cytokine related and include interleukin-2 receptor binding (Strength = 2.89), cytokine receptor activity (Strength = 1.91), and cytokine receptor binding (Strength = 1.47). The top CC is the protein complex involved in cell adhesion (Strength = 2.05). One metal ion BP term is identified about the positive regulation of cytosolic calcium ion concentration (Strength = 1.3). The hub genes make it very clear that the immune system is an important process in this data set, with how greatly comprised of immune related terms this set of GO terms is. These hub genes and their interactions can be seen in Figure 8.

#### Down-regulated

In the bulk down-regulated DEGs many terms are related to the neurocognitive pathways, but also include immune related terms (Supplementary Table 2). The top BP is the positive regulation of stem cell differentiation (Strength = 0.96) and followed by parasympathetic nervous system development (Strength = 0.88). This is followed by autonomic nervous system development (Strength = 0.74), regulation of dopamine secretion (Strength = 0.73), and synaptic signaling (Strength = 0.3). Additionally, the BP terms neuron development (Strength = 0.24), neuron differentiation (Strength = 0.22), and generation of neurons (Strength = 0.21) are found in this down-regulated set of DEGs. Amongst the CC terms, the strongest is the GABA-ergic synapse (Strength = 0.55) and is followed by the synaptic membrane (Strength = 0.35). This follows the identified pattern seen in Cruceanu and Iwamoto where neurocognitive terms related to neurotransmitter secretion and neuronal health are in the down-regulated set of DEGs.

Additionally, there were two BP terms related to the immune system in this set which were the regulation of interleukin-2 production (Strength = 0.63) and cytokine production (Strength = 0.46). There was also the BP term regulation of ion transport (Strength = 0.24) and MF term calcium ion binding (Strength = 0.28), which we have observed in the other data sets to primarily be found in down regulated DEGs of people with BD.

In the hub genes, $\frac{43}{65}$ terms are directly related to the immune system (Supplementary Table 2). The strongest BP term is the negative regulation of T-helper cell differentiation (Strength = 2.59), followed by positive regulation of regulatory T cell differentiation (Strength = 2.45). After these, the BP terms include the positive regulation of interleukin-17 production (Strength = 2.36), negative regulation of interleukin-2 production (Strength = 2.23), and many more terms related to T cells and cytokine production. This contrasts some of the results in the other data sets, such as the Iwamoto data set, where cytokine production pathways were found in the up-regulated DEGs and not the down-regulated DEGs.

#### Up-regulated

In the bulk up-regulated DEGs, there are several neurocognitive related terms related to neuronal communication (Supplementary Table 2). The top BP is dopamine uptake involved in synaptic transmission (Strength = 1.41). The top CC is the cell-cell junction (Strength = 0.36), followed by the synapse and axon (Supplementary Table 2). These terms contrast some of the results found in the down-regulated DEGs in this data set, however, there are fewer related terms to the neurocognitive pathways with 19 neurocognitive terms in the down-regulated set and only 7 in the up-regulated set.

The hub genes show several immune system related terms, the strongest BP term of which is the negative regulation of acute inflammatory response (Strength = 2.48). This is followed by the negative regulation of T cell differentiation (Strength = 1.94), negative regulation of immune response (Strength = 1.58), and regulation of leukocyte proliferation (Strength = 1.39). These show the opposite results compared to the down-regulated DEGs hub genes, but in the up-regulated DEG hub genes, only $\frac{7}{71}$ are immune related compared to the $\frac{43}{65}$ found in the hub genes of down-regulated DEGs in the Matigian data set (Supplementary Table 2).

### 0.11 Savitz Study - PBMC Bipolar vs Control

#### Bulk

The bulk set of DEGs contains terms related to the immune system, RNA, neurocognitive, and metal ion pathways (Supplementary Table 2). The top BP term is the negative regulation of synaptic transmission, dopaminergic (Strength = 2.65), followed by dopamine biosynthetic process (Strength = 2.13) and positive regulation of neuron death (Strength = 1.31). Once again, terms related to dopamine and neuron death have been identified as in the Cruceanu and Iwamoto data sets. Immune related terms include the BP terms positive regulation of fever generation (Strength = 2.29), response to glucocorticoid (Strength = 1.26), and cytokine-mediated signaling pathway (Strength = 0.78). Glucocorticoids reduce inflammation and suppress the immune system. Contrasting each other, we found 2 RNA BP terms which were the negative regulation of transcription by RNA polymerase II (Strength = 0.72) and positive regulation of transcription by RNA polymerase II (Strength = 0.62). Finally, two metal ion BP terms were identified which are the cellular response to metal ion (Strength = 1.24) and response to metal ion (Strength = 1.04).

**Figure 9.**
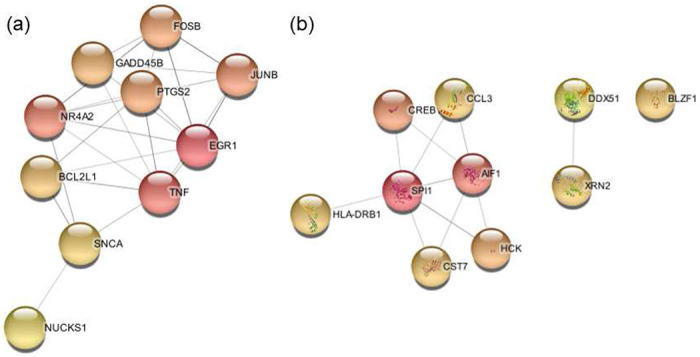
**A)** Whole STRING gene interaction network of the Savitz Bipolar vs Control data set’s top 10 key hub genes with LogFC > |0.5|. Confidence score > 0.4 **B)** Whole STRING gene interaction network of significantly differentially expressed genes with LogFC > |0.5|. These are the top 10 hub genes in the STRING network.

The hub genes are composed in a similar distribution as the bulk set of DEGs. The hub genes and their interactions can be seen in Figure 9. For the immune related BP terms, it includes microglial cell activation (Strength = 2.09), interleukin-1 beta production (Strength = 1.85), positive regulation of chemokine production (Strength = 1.81), positive regulation of inflammatory response (Strength = 1.61), and cytokine-mediated signaling pathway (Strength = 1.16). As in the before discussed data sets, terms related to the activation of immune cells and the production of cytokines were found. There are several RNA related terms, which are the BP terms positive regulation of pri-miRNA transcription by RNA polymerase II (Strength = 1.99), negative regulation of transcription by RNA polymerase II (Strength = 1.12), and positive regulation of transcription by RNA polymerase II (Strength = 0.97). There is also the top MF term which is DNA-binding transcription activator activity, RNA polymerase II-specific (Strength = 1.34). There are two BP terms related to metal ions which are the cellular response to calcium ions (Strength = 1.66) and the cellular response to metal ions (Strength = 1.61).

There are several neurocognitive related terms, with the strongest BP term being the negative regulation of synaptic transmission, dopaminergic (Strength = 3.12). This is followed by the BP terms dopamine biosynthetic process (Strength = 2.59), positive regulation of neuron death (Strength = 1.78), regulation of synaptic transmission, glutamatergic (Strength = 1.77), and regulation of neuron apoptotic process (Strength = 1.44). These are consistent with the observations in the bulk set of DEGs in this data set. One of the notable hub genes identified is TNF-*Δ* which has been consistently reported to have significant up regulation in bipolar patients compared to controls^22, 23, 28^.

#### Down-regulated

No significantly enriched GO terms were identified in the set of down-regulated DEGs or their hub genes.

#### Up-regulated

The top BP GO term is the negative regulation of synaptic transmission, dopaminergic (Strength = 2.75), followed by other neurocognitive related BP terms such as the dopamine biosynthetic process (Strength = 2.23) and positive regulation of neuron death (Strength = 1.42). This continues the pattern where up-regulated DEGs contain GO terms related to a reduction in synaptic transmission and an increase in neuron death. Additionally, there are several immune system related terms (Supplementary Table 2). The BP terms negative regulation of cytokine production involved in immune response (Strength = 1.83), response to glucocorticoid (Strength = 1.36), and inflammatory response (Strength = 0.92) are found. There are also several RNA related terms such as the contradicting BP terms negative of transcription by RNA polymerase II (Strength = 0.82) and positive regulation of transcription by RNA polymerase II (Strength = 0.68). There are two metal ion BP terms which are the cellular response to metal ion (Strength = 1.34) and the response to metal ion (Strength = 1.14).

The hub genes show a similar composition of pathways (Supplementary Table 2). The top BP is the negative regulation of synaptic transmission, dopaminergic (Strength = 3.12), followed by the dopamine biosynthetic process (Strength = 2.59), positive regulation of neuron death (Strength = 1.78), regulation of synaptic transmission, glutamatergic (Strength = 1.77), and regulation of neuron apoptotic process (Strength = 1.44). The immune system related terms include the BP terms microglia cell activation (Strength = 2.09), positive regulation of interleukin-1 beta production, positive regulation of chemokine production (Strength = 1.81), and positive regulation of inflammatory response (Strength = 1.61). These terms are consistent with an up-regulation in cytokines and inflammation as was seen in the Clelland and Iwamoto data sets. This set also includes the RNA BP terms positive regulation of pre-miRNA transcription by RNA polymerase II (Strength = 1.99), negative regulation of transcription by RNA polymerase II (Strength = 1.12), and positive regulation of transcription by RNA polymerase II (Strength = 0.89). The top MF is the DNA-binding transcription activator activity, RNA polymerase II-specific (Strength = 1.24). The BP metal ion terms include only the cellular response to metal ions (Strength = 1.61).

### 0.12 Savitz Study - PBMC Bipolar Type 1 vs Bipolar Type 2

#### Bulk

In the bulk set of DEGs, there is only one significantly enriched GO term which is the BP regulation of developmental process (Strength = 0.44).

The hub genes are composed primarily of immune system related genes, making up $\frac{6}{8}$ GO terms in the set (Supplementary Table 2). This includes the BP terms the cellular response to interferon-gamma (Strength = 1.69), regulation of myeloid leukocyte differentiation (Strength = 1.69), regulation of inflammatory response (Strength = 1.35), cellular response to cytokine stimulus (Strength = 1.06), and regulation of immune system process (Strength = 0.96). These terms are mostly related to cytokines and myeloid cells. The protein interaction network of these hub genes can be seen in Figure 9.

#### Down-regulated

There were two enriched GO terms in the full set of down-regulated DEGs, but zero enriched terms for their hub genes. The two GO terms that were enriched were the BP term regulation of molecular function (Strength = 0.43) and the MF term molecular function regulator (Strength = 0.52). There were no significantly enriched GO terms in the hub genes of this set of down-regulated DEGs.

#### Up-regulated

No significantly enriched GO terms were identified within the bulk set of up-regulated DEGs, but its hub genes were enriched for immune system terms primarily related to cytokine signaling and response, making up $\frac{3}{4}$ of the GO terms. The top BP term is the negative regulation of gene expression, epigenetic (Strength = 1.76) and is followed by the terms regulation of myeloid cell differentiation (Strength = 1.48), cytokine-mediated signaling pathway (Strength = 1.16), cellular response to cytokine stimulus (Strength = 1.06), and regulation of immune system process (Strength = 0.96).

Since the down-regulated DEGs only had significant GO terms related to the regulation of MF, these results could imply that the enriched GO terms related to the immune system are primarily composed of up-regulated genes. It could then indicate that these inflammatory genes tend to be more up-regulated in individuals with BD 1 versus individuals with BD 2.

### 0.13 Vawter Study - Anterior Cingulate Bipolar Type 1 vs Control

#### Bulk

Only 2 GO terms are identified in the full set of DEGs, both CC terms. The top GO term is the CC transcription factor AP-1 complex (Strength = 2.91). An AP-1 transcription factor regulates gene expression in response to a variety of stimuli, including cytokines, growth factors, stress, and bacterial and viral infections. The other CC term is the nuclear chromosome (Strength = 0.89).

In the hub genes, the CC term transcription factor AP-1 complex is also enriched (Strength = 2.99). Additionally, a RNA related term RNA polymerase II cis-regulatory region sequence-specific DNA binding (Strength =1.16) is also enriched.

#### Down-regulated

Both the bulk down-regulated DEGs and their hub genes are enriched for one term, which is the transcription factor AP-1 complex (Strength = 2.99).

#### Up-regulated

There are zero significantly enriched GO terms in the up-regulated set of DEGs or its hub genes.

### 0.14 Witt Study - Blood Bipolar Type 1 vs Control

#### Bulk

Most terms are related to muscle movement, kinases, and membrane structure (Supplementary Table 2). Due to the size of the data, we used Enrichr to analyze the pathways. The top BP term related to neurocognitive pathways is the transport across the blood-brain barrier (Odds Ratio = 2.245) although this is indirectly related. This is followed by axonogenesis (Odds Ratio = 1.589) which is more directly related to the pathway of interest. There is additionally the RNA related MF term adenyl ribonucleotide binding (Odds Ratio = 1.491). There are three metal ion related terms which are the BP terms metal ion transport (Odds Ratio = 2.569), calcium ion transport (Odds Ratio = 1.984), and the MF term voltage-gated calcium channel activity (Odds Ratio = 3.527). Once again, calcium appears in these metal ion terms as in the Cruceanu, Iwamoto, and Logotheti data sets.

We analyzed these hub genes using STRING. In the hub genes, the large majority of these terms were related to cellular processes, especially mitosis (Supplementary Table 2). There is a strongly enriched BP term called the positive regulation of mitochondrial ATP synthesis coupled electron transport (Strength = 2.89). The top MF is histone kinase activity (Strength = 2.56) and the top CC is the cyclin b1-cdk1 complex (Strength = 3.29).

#### Down-regulated

The down-regulated set of DEGs contains terms related to the cell cycle and regulation of transcription. The top BP term is the ciliary basal body-plasma membrane docking (Odds Ratio = 2.729). This is followed by the negative regulation of transcription, DNA-templated (Odds Ratio = 1.469) and the regulation of transcription by RNA polymerase II (Odds Ratio = 1.328).

The hub genes were analyzed using STRING and primarily enriched for RNA related terms and compose $\frac{6}{7}$ of the terms (Supplementary Table 2). The only BP term is ribosome biogenesis (Strength = 1.6). The top MF term is RNA helicase activity (Strength = 2.12), followed by catalytic activity acting on RNA (Strength = 1.5), RNA binding (Strength = 1.07), and purine ribonucleoside triphosphate binding (Strength = 0.81). The only CC is the nucleolus (Strength = 1.23).

#### Up-regulated

This up-regulated set of DEGs, analyzed with Enrichr, contains terms related to the immune system, ribosomal, neurocognitive, metal ion, and ATP related pathways (Supplementary Table 2). The top immune system BP is the negative regulation of interleukin-10 production (Odds Ratio = 5.514), followed by antigen processing and presentation of peptide antigen via MHC class I (Odds Ratio = 3.612), antigen processing and presentation of exogenous peptide antigen via MHC class II (Odds Ratio = 2.079), and neutrophil activation involved in immune response (Odds Ratio = 1.689). This analysis identified terms related to MHC class I and class II which were also seen in the Chen and Liu data set. The RNA related MF term adenyl ribonucleotide binding (Odds Ratio = 1.534) was also identified. The BP terms related to metal ions were potassium ion homeostasis (Odds Ratio = 6.021), sodium ion homeostasis (Odds Ratio = 4.514), and calcium ion transport (Odds Ratio = 2.011). The MF terms include calcium ion binding (Odds Ratio = 1.513), transition metal ion binding (Odds Ratio = 1.511), and zinc ion binding (Odds Ratio = 1.492). The top CC term is the sodium channel complex (Odds Ratio = 7.745). There is additionally the MF term ATP binding (Odds Ratio = 1.544) and CC term mitochondrial membrane (Odds Ratio = 1.340).

The neurocognitive terms identified included the BP term regulation of dendritic cell differentiation (Odds Ratio = 9.022), positive regulation of neuron death (Odds Ratio = 3.143), and axonogenesis (Odds Ratio = 1.816). The MF terms included very high Odds Ratio terms such as the kinate-selective glutamate receptor activity (Odds Ratio = 75015), nerve growth factor binding (Odds Ratio = 75015), and is followed by the terms neurotrophin binding (Odds Ratio = 21.045) and neurotransmitter receptor activity involved in regulation of postsynaptic membrane potential (Odds Ratio = 4.889). The CC terms include the axon (Odds Ratio = 1.833) and neuron projection (Odds Ratio = 1.503). As in the Iwamoto data set, the term for neuron cell death is found in this set of up regulated DEGs.

The hub genes were once again analyzed using STRING. In the hub genes, the same mitochondrial GO term is identified as in the bulk hub genes. This is also the top BP term and is called the positive regulation of mitochondrial ATP synthesis coupled electron transport (Strength = 2.89). The majority of the remaining terms are related to mitosis and the cell cycle process. There is also the immune related CC term host cell nucleus (Strength = 1.49).

### 0.15 Brain Tissue Data Set Gene Comparison

**Figure 10.**
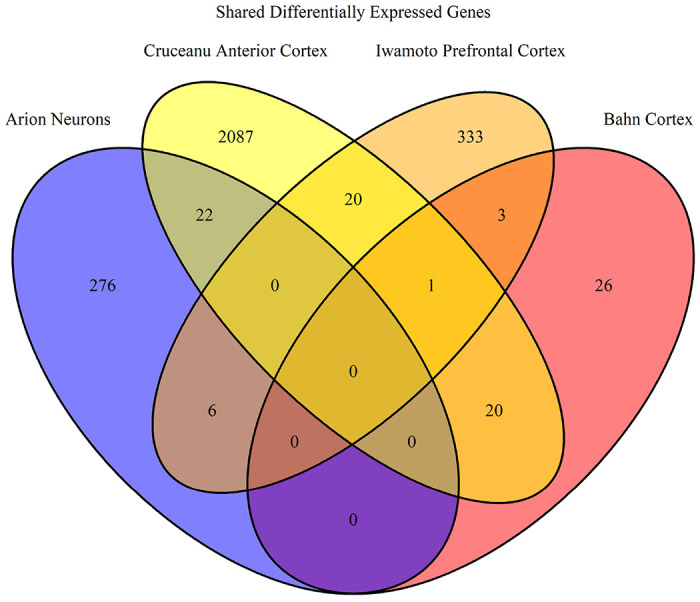
Brain Data Sets DEGs Venn Diagram Venn diagram of the differentially expressed genes from brain tissue derived data sets of Arion, Bahn, Cruceanu, and Iwamoto.

This comparison of the Arion, Bahn, Iwamoto, and Cruceanu data sets is conducted to compare the common genes between these data sets composed of human brain tissue. Zero significant genes were shared between all four data sets (Figure 10). Only 1 gene, MT1X, is shared between 3 of the data sets (Bahn, Cruceanu, and Iwamoto). This gene enables copper and zinc ion binding activity.

### 0.16 Non-Medicated Samples Gene Comparison

**Figure 11.**
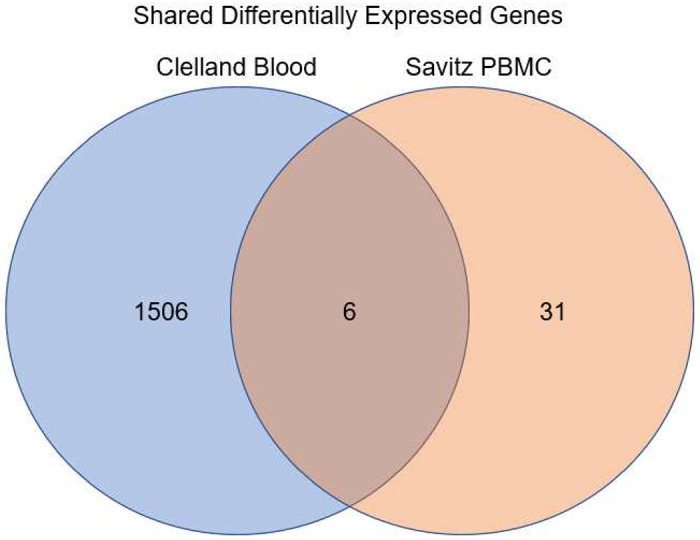
Non-Medicated Data Sets DEGs Venn Diagram Venn diagram of the differentially expressed genes from Clelland Blood Never Medicated Bipolar Type 1 vs Savitz PBMC Bipolar vs Control.

This compares the Clelland and Savitz Bipolar vs Control data sets since these data sets are composed of blood samples from patients who were not taking medication for BD. Six overlapping genes were observed: SNCA, NUCKS1, ADM, GADD45B, RHOB, and SNORD3D (p < 0.05, LogFC > |0.5|) (Figure 11).

We note the gene **SNCA** which encodes the protein *α*-synuclein and is most abundant in the brain, primarily in presynaptic terminals which release neurotransmitters^75^. It is found throughout the rest of the central nervous system and can be found in other tissues. It is suggested that it is involved in the regulation of dopamine release, movement of microtubule structures, and metal ion binding activity. It has been heavily implicated in Parkinson’s disease as well as in Alzheimer’s disease and Lewy body dementia^75^.

### 0.17 Blood Data Sets Gene Comparison

**Figure 12.**
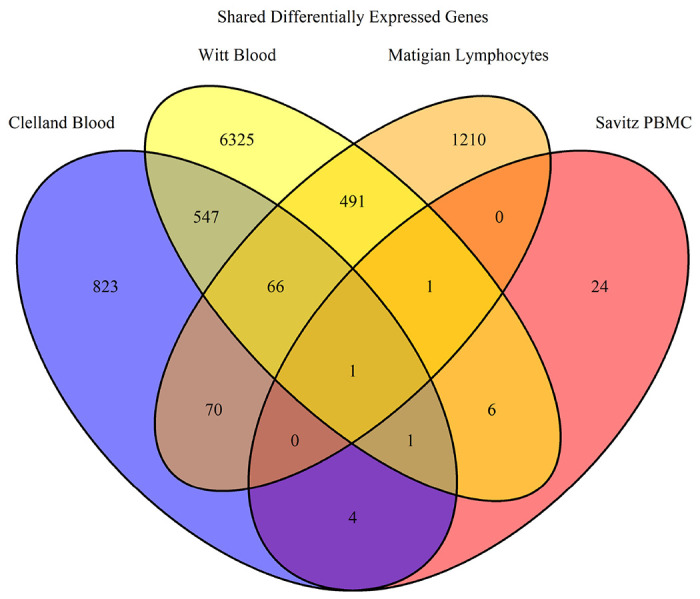
Blood Data Sets DEGs Venn Diagram Venn diagram of the differentially expressed genes from the blood data sets of Clelland, Matigian, Savitz, and Witt.

This is a comparison of the Clelland, Matigian, Savitz, and Witt data sets which used samples derived from blood (Figure 12). The single common gene is **SNCA**. Since SNCA was found to be a significantly differentially expressed gene in both medicated and non-medicated data sets, it could potentially serve as a biomarker that can be identified regardless of the individual’s treatment status for psychiatric disorders. SNCA was one of the key hub genes of Savitz BD vs Control. Additionally, SNCA was identified in blood samples which makes it a lot more practical as a biomarker. We report the LogFC for SNCA from data sets where LogFC > |0.5| and p < 0.05:

- Savitz BD vs Control: 0.632
- Clelland BD 1 vs Control: −0.86043375
- Matigian BD vs Control: 2.26207627
- Witt BD 1 vs Control: 0.608
- Cruceanu BD vs Control: −0.526488911

The differential gene expression of SNCA varies between data sets, instead of being consistently down-regulated as reported in other studies^76^. This could be explained by treatment status, as it has been identified that lithium treatment increases the expression of *α*-synuclein^77^. Thus, the Clelland data set where SNCA is down-regulated could be explained because the data set is composed entirely of individuals who never received drug treatment for BD. However, further in vivo investigation is necessary to validate these observations and understand the mechanisms occurring in the blood and brain tissue. Additionally, a reduced expression of SNCA could potentially lead to a decrease in neurotransmitter release which is consistent with the results identified in this study.

### 0.18 BD 1 Data Sets Gene Comparison

**Figure 13.**
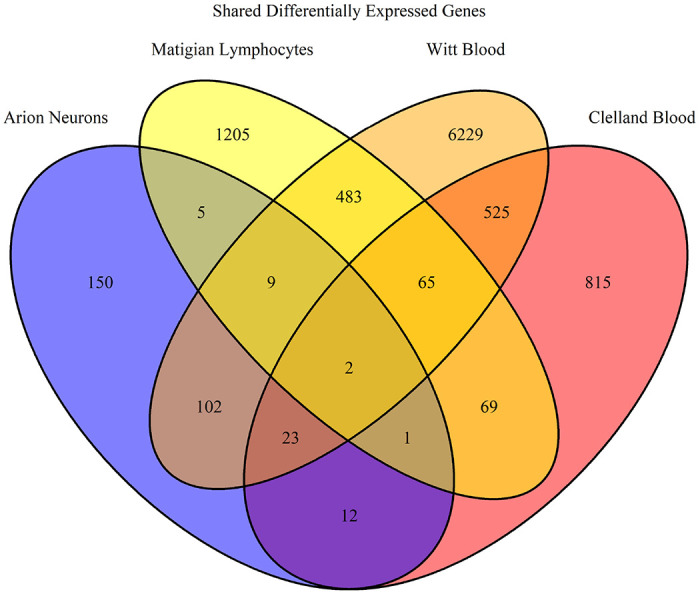
Venn Diagram BD Type 1 Arion Clelland Matigian Witt DEGs Venn diagram of the differentially expressed genes from the Arion, Clelland, Matigian, and Witt data sets.

This is a comparison of the Arion, Clelland, Matigian, and Witt Bipolar Type 1 data sets (Figure 13). These are the data sets containing BD 1 and either blood or brain samples. The 2 common genes are SPP1 and MAP4K4. SPP1, also known as osteopontin, is a cytokine that recruits cells to sites of inflammation and is involved in anti-apoptosis, promoting bone remodeling, and inhibiting IL-10 production. SPP1 is found primarily in bone, but also in macrophages, neutrophils, dendritic cells, microglia, T cells, and B cells. It is related to cancer, autoimmune diseases, and Parkinson’s Disease. Interestingly, Valproic acid, a treatment for Bipolar Disorder and epilepsy, was found in vitro to increase SPP1 expression in stromal cells^78^. MAP4K4 promotes and mediates TNF-*α* signaling, promotes TNF-*α* expression, and TNF-*α* can promote MAP4K4 expression^79,80^. This gene also is likely involved in the negative regulation of insulin-dependent glucose transport due to its effect on TNF-Δ. The gene is mostly implicated in cancer. These results support the identification of enriched inflammation related GO terms. While these results do not specify if these genes are expressed differently in BD 1 versus BD 2, it does give further information for BD as a whole.

## Discussion

We analyzed 17 different gene expression data sets for significantly enriched genetic pathways and identified five genetic pathways that were found across this data. 15 data sets were examined who had more than 10 DEGs (Table 4). This critical summary shows that the data sets were very enriched for the immune system and RNA related terms. Specifically, 12 were enriched for immune system related terms, 12 were enriched for RNA related terms, nine were enriched for neurocognition related terms, nine were enriched for metal ion related terms, and five were enriched for ATP related terms.

Many of these data sets do not share exactly the same GO terms, but they often share key genetic pathways or the direction in which genetic pathways are affected. Additionally, they do not share many of the differentially expressed genes. This would best fit the idea that many genes are affecting the same pathway, but in different parts of the pathway. It may explain the variation in the cytokine studies where proteins or immune factors are not consistently found at a significantly different expression level between patients and controls.

Except for the Arion and Kim data sets which were composed of neurons, enriched terms related to the immune system appear in all of the 15 data sets. The identification of hub genes related to TNF-*α* and the interferon system are consistent with the reported association of TNF-*α* and IFN-*γ* with BD 2^7^. Many of the identified GO terms were related to the proliferation, differentiation, and activation of immune cells. Additionally, many of the identified genes are related to the interleukin system and cytokines as a whole. Numerous cytokine production-related terms with a high degree of Strength were found in up-regulated DEGs of the Clelland and Iwamoto data sets. An increase in cytokine levels is consistent with prior reports^22–25^. Some data sets, such as the Logotheti and Matigian data sets instead found some interleukins and cytokines in their down-regulated set of DEGs. This could be explained as an overall response by the immune system to maintain homeostasis or could indicate a much more complex interaction than just cytokine levels being elevated. Further studies in BD should examine the exact mechanisms by which these immune components interact within BD.

RNA related GO terms were identified in the majority of data sets. Ribosomal structure-related GO terms appear frequently in these data set analyses, and often refer to earlier stages of ribosomal genesis. Many of the BP and MF terms are related to the splicing of rRNA and mRNA. Additionally, many cellular components were in the ribosome, specifically the pre-ribosome, and in the nucleus. It may indicate that the effects of differentially expressed genes in BD affect RNA, mRNA, and rRNA early in the protein synthesis process while the messages encoding the factors are still in the transcriptional stage. This also matches that many of the identified BP terms were related to transcript modifications. For the most part, data sets identified these transcription-related processes as down-regulated in individuals with BD. These include terms related to cleavage, binding, and metabolic processes. This is most notable in the Clelland data set where almost the entirety of RNA related terms were found in the down-regulated DEGs. In the Arion BD 1 vs Control comparison, the only significant GO terms related to RNA were found to be down-regulated. In the Arion BD NOS vs Control, most terms related to RNA processing were in the down-regulated set of DEGs, although some were found in the up-regulated DEGs hub genes. RNA physiology, especially at the transcriptional stage, seems to have some relevant role to BD which needs to be further investigated.

Neurocognition related GO terms were notably divided by the direction of differentially expressed genes. In the Bahn, Cruceanu, Iwamoto, and Matigian data sets, terms related to neuronal health, synaptic signaling, and neurotransmitter secretion were found specifically enriched in down-regulated DEGs. In the Iwamoto and Witt data set, terms related to neuron cell death and apoptosis were found in the up-regulated DEGs. The neurotransmitters dopamine, glutamate, and GABA were commonly found in down-regulated sets of DEGs. Additionally, GO terms related to the secretion of these neurotransmitters were also found in sets of down-regulated DEGs. Many of the cellular components identified in this data pertain specifically to the synapse, which is the key site of neuron-to-neuron communication. Given the enriched GO terms related to the secretion of neurotransmitters, synaptic signaling, and overall neuronal health were found in the down-regulated sets of DEGs, these results are consistent with the idea that neuronal activity is reduced in individuals with BD.

Metal ion related GO terms appeared in roughly half the data sets. Notably, calcium ion related terms seem to appear far more commonly than the other metal ions. Calcium plays a number of roles in brain function. Possibly, calcium ions were commonly identified due to their role in exocytosis; the release of neurotransmitters from vesicles into the synaptic cleft enabling communication with other neurons. Calcium ions control the release of neurotransmitters from synaptic vesicles by binding to synaptotagmins^81^. They are also fundamental to the propagation of action potential in neurons, as well as a large part in activating pro-inflammatory processes in glial cells. Calcium is also a significant molecule in cell death pathways. Calcium ions made up the majority of metal ion terms in the Cruceanu, Iwamoto, and Logotheti data sets. Further, these calcium dependent terms were almost entirely found in the set of down-regulated DEGs. Notably, a meta-analysis of calcium in BD finds intracellular calcium to be increased in BD in a number of cells^82^. Additionally, these cells from people living with BD tend to have increased responses to calcium^82^. Other studies analyzing the Bahn data set found similar results with enriched BP GO terms in the set of up-regulated DEGs^29^. This consistency is warrants further investigation into the mechanistic role of calcium ions in BD.

Finally, ATP related terms were found in roughly a third of data sets. These terms were related to ATPase, ATP binding, cellular respiration, and other terms related to mitochondria. In the Arion BD NOS vs Control data set, ATPase binding was found in the down-regulated set of DEGs which implies a lower amount of energy available for cells. However, most ATP related terms were found in up-regulated sets of DEGs, such as in the Cruceanu, Iwamoto, and Witt data sets. In those up-regulated data sets, there was an enrichment of terms related to ATP binding and ATP metabolic processes.

We identify SCNA as a potential novel biomarker that has not been previously investigated in BD. SNCA was found consistently in data sets of both non-medicated subjects and blood samples which included specimen from medicated individuals. This makes the SNCA worth further investigation with regard to its potential to identify BD in both treated and untreated patients and because it can be detected in blood samples. The function of SNCA is believed to be in the regulation of the release of dopamine but it is also potentially related to metal ion binding. These functions are directly linked to several of the pathways we identified as important in this analysis. Other studies have demonstrated that elevated levels of *α*-synuclein lead to worsening neuronal health^83^.

Very limited research has been done on the relationship between SNCA and Bipolar Disorder. Gibbons et al. did not find significant differences in mRNA expression levels between SNCA in BD and control from samples taken from Brodmann’s Area 24 (anterior cingulate)^84^. However, another study reports that lithium treatment in a chronic 1-methyl-4-phenyl-1,2,3,6-tetra-hydropyridine (MPTP) mouse model, reduced the loss of nigral neurons while also increasing the expression of *α*-synuclein in substantia nigra and striatum^77^. Overall, the authors were able to reduce cognitive deficits through lithium treatment.

Additionally, a study conducted by Gray et al. identified the levels of *α*-synuclein to be decreased in individuals with BD 1 compared to controls^76^. The authors propose that the reduction in *α*-synuclein levels could inhibit synaptic function by disrupting the release of neurotransmitters^76^. In our study, we have consistently identified a down-regulation in genes related to neurotransmitter release, especially in synaptic transmission, which provides evidence for their hypothesis. Additional studies found less *α*-synuclein in individuals with Bipolar Disorder compared to age-matched controls^85, 86^.

While we cannot say with certainty at this time that SNCA can be used as a biomarker for BD, it seems a gene worth analyzing in blood samples when attempting to diagnose BD. Thus, future studies should investigate the role of SNCA in BD as there are currently few studies that include it.

Additionally, the study conducted by Witt et al. looked at different samples collected from BD patients in manic versus euthymic states. In their study, 22 genes where differentially expressed between manic and euthymic state patients^52^. In their study, comparing the different states against controls, they also identify genes that are relevant to RNA related KEGG pathways and disease related KEGG pathways such as Parkinson’s and Alzheimer’s^52^.

We acknowledge that there are limitations to our data analysis. More data needs to be collected that specifies the subtypes of Bipolar Disorder, especially for BD 2 and Cyclothymic disorder. The large majority of BD data we discovered consisted of BD 1 samples. Not all data sets are equal, with different data sets containing different numbers of samples which can confound analysis. This is especially true for medication where data sets contained samples that were taking different medications.

The techniques used to analyze the key hub genes of these data sets involved analyzing the hub genes of protein-protein interaction networks as calculated using the MCC score. Due to this, STRING cannot include non-protein coding genes in its network. For example, the Vawter data set has 44 DEGs but only 12 proteins are recognized by STRING (Table 1). Thus, there could be key non-coding genes involved in Bipolar Disorder that couldn’t be captured using this technique.

Going forwards, researchers should aim to differentiate their Bipolar Disorder samples by subtype. Even if they are not directly studying questions related to subtype differences, doing so can help the overall field differentiate the genes that underlie these subtypes. Identifying these specific factors can improve our understanding of the genetic characteristics of BD and can eventually lead to specific biomarker identification for diagnosis and treatment.

Ultimately, this study identifies key genetic pathways related to BD as well as identifies a potential novel biomarker, SNCA. Further research should be done to look at the role of genes in the immune and RNA pathways at a mechanistic level so we can better understand the pathology of BD and uncover additional new biomarkers.

## Supplementary Material

1

## Figures and Tables

**Table 1.** Number of Significantly Differentially Expressed Genes in Each Data Set Information on the total number of significant DEGs and their direction of regulation that have a p value < 0.05 and LogFC > |0.5|. Additionally, we report the number of DEGs that are recognized by STRING and used to generate the protein-protein interaction network for each data set. Genes may not be recognized by STRING due to a difference in naming convention by the probes used to identify the gene, or the gene may represent a non-coding gene.

Data set	Down-regulated	Up-regulated	Total	Recognized by STRING

Abdolmaleky BD vs Control	0	0	0	0

Arion BD 1 vs Control	204	100	304	296
	
Arion BD NOS vs Control	462	792	1254	1223
	
Bahn BD vs Control	48	2	50	47

Bousman BD vs Control	95	43	138	65
	
Chen BD vs Control	3	4	7	7

Chen and Liu BD vs Control	2	0	2	2
	
Clelland BD 1 vs Control	1183	329	1512	1308
	
Cruceanu BD vs Control	1535	615	2150	1358

De Baumont BD vs Control	0	0	0	0
	
Hilscher BD vs Control	16	6	22	15

Iwamoto BD vs Control	161	202	363	325

Kim BD 1 vs Control	392	676	1068	866
	
Liu BD vs Control	2	1	3	3

Logotheti BD 1 vs Control	364	259	623	553
	
Matigian BD 1 vs Control	1057	782	1839	1418
	
Savitz BD 1 vs BD 2	33	33	66	61

Savitz BD vs Control	7	30	37	29
	
Vawter BD 1 vs Control	42	2	44	12

Witt BD 1 vs Control	2441	4997	7438	7208

**Table 2.** Demographics Basic information about each data sets’ GEO Accession number, number of samples, biological sample material, BD medication status, and sequencing platform. More detailed information about each data set can be found in the supplementary information.

Data set	GEO Accession	Number of Samples	Biological Material	BD Medication	Sequencing Platform

Abdolmaleky	GSE120341	35 BD, 35 Control	Prefrontal Cortex	Yes	GPL8490
		
Arion	GSE87610	17 BD 1, 2 BD NOS , 19 Control	Dorsolateral Prefrontal Cortex	Yes	GPL13667
		
Bahn	GSE5392	40 BD, 42 Control	Orbitofrontal Cortex, Dorsolateral Prefrontal Cortex	Yes	GPL96
		
Bousman	GSE18312	9 BD, 8 Control	PBMC	Yes	GPL5175

Chen	GSE35977	45 BD, 50 Control	Parietal Cortex	Yes	GPL6244
		
Chen and Liu	GSE35978	82 BD, 100 Control	Cerebellum, Parietal Cortex	Yes	GPL6244

Clelland	GSE46449	3 Never Medicated BD 1, 25 Control	Peripheral Blood Leukocytes	No	GPL570
		
Cruceanu	N/A	13 BD, 13 Control	Anterior Cortex	Yes	GPL11154
		
De Baumont	GSE62191	29 BD, 30 Control	Frontal Cortex	Yes	GPL4133
		
Hilscher	GSE66196	29 BD, 30 Control	Frontal Cortex	Yes	GPL4133
		
Iwamoto	GSE12654	11 BD, 15 Control	Prefrontal Cortex	Yes	GPL8300
		
Kim	GSE74358	4 BD 1, 4 Control	iPSCs differentiated into neuroprogenitors then neurons	Yes	GPL570
		
Liu	GSE35974	37 BD, 50 Control	Cerebellum	Yes	GPL6244

Logotheti	GSE69486	10 BD 1, 5 Control	Skin Fibroblasts	Yes	GPL10558
		
Matigian	GSE7036	3 BD 1, 3 Control	Lymphocytes	Yes	GPL570

Savitz	GSE39653	4 BD 1, 4 BD 2, 24 Control	PBMC	No	GPL10558
		
Vawter	GSE78246	9 BD 1, 11 Control	Anterior Cingulate Cortex	Yes	GPL5175

Witt	GSE46416	11 BD 1, 10 Control	Blood	Yes	GPL11028

**Table 3.** **All Hub Genes** The 10 hub genes for each bulk data set comparison and their direction of regulation. If the gene is not directly in the list of DEGs with a p value < 0.05 and LogFC > |0.5| then it is listed under **Indirect** and was a related gene that STRING identified as a key hub gene.

Data set	Down-regulated	Up-regulated	Indirect

Abdolmaleky BD vs Control	N/A	N/A	N/A
	
Arion BD 1 vs Control	KCNJ9, RRS1, FCF1, FTSJ3, LTV1, EMG1, GAR1, NHP2	WDR36, POLR1B	N/A

Arion BD NOS vs Control	SKIV2L2, GTPBP4, GNL3, FTSJ3	BRIX1, UTP14C, WDR36, NOP14, TBL3, KIAA0020	N/A
	
BahnBD vs Control	SST, TAC1, VSNL1, ATP2B2, NPY, GABRG2, SNAP25, GABRA1, SYT1, NEFL	N/A	N/A

Bousman BD vs Control	HDC, MS4A2, FCER1A, CPA3	HIST1H3A, HIST1H4D, HIST1H4B	HIST1H3D, HRAS, HIST1H4E
	
Chen BD vs Control	CYBB, SST, C3	GJB6, RANBP3L, SLC14A1, WIF1	N/A
	
Chen and Liu BD vs Control	HLA-DRA, HLA-DQA1	N/A	N/A
	
Clelland BD 1 vs Control	HNRNPA2B1, HNRNPM, SFPQ, SRSF10, TAF15, TARDBP	HNRNPA1, HNRNPC, PTBP1, RBM14	N/A

Cruceanu BD vs Control	N/A	RPS21, RPL30, RPL23A, RPL37A, RPL38, RPL31, RPS18, RPL21, RPS27, RPS25	N/A
	
De Baumont BD vs Control	N/A	N/A	N/A
	
Hilscher BD vs Control	GAGE12F, GAGE12G, EYA2, ZNF595, TDRD9, BCHE	PSPH, KLRC2, KLRC1	GPR128

Iwamoto BD vs Control	IL7, CD4, IL1RN	CSF2, INS, VCAM1, IFNB1, IL4, TP53, IFNG	N/A
	
Kim BD 1 vs Control	COL1A1, COL6A2, COL6A3, COL5A1, COL14A1, COL12A1, COL15A1, COL3A1, COL1A2, COL5A2	N/A	N/A

Liu BD vs Control	ZP2, VEPH1	LINGO2	N/A

Logotheti BD 1 vs Control	PPARG, INS, CACNG6, CATSPERD, RYR1, CATSPER2, CACNA1C, TEX40, CACNA2D3	PDE4B	N/A
	
Matigian BD 1 vs Control	FOXP3, IL12RB1, GATA3, IL2, IL18, CD4, ITGAX	CTLA4, CCRL2, IL7R	N/A

Savitz BD vs Control	NUCKS1	BCL2L1, NR4A2, PTGS2, FOSB, GADD45B, SNCA, JUNB, EGR1, TNF-*α*	N/A
	
Savitz BD 1 vs BD 2	CREB1, CCL3, CST7, XRN2, BLZF1, DDX51	AIF1, HCK, SPI1, HLA-DRB1	N/A

Vawter BD 1 vs Control	DDX3X, DUSP1, FOS, JUNB, NR4A1, USP9X, HLA-DPA1	N/A	ZFP42, DCLRE1A, HNRNPU
	
Witt BD 1 vs Control	N/A	CDK1, CENPF, NCAPG, CCNA2, BUB1, BUB1B, AURKB, MELK, CCNB1, ASPM	N/A

**Table 4.** Summary of Enriched Gene Pathways This table shows which data sets contained GO terms that were part of one of the five key pathways. Green cells denote that the pathway was found in the specified data set comparison while white cells denote that the pathway was not found.

Data set	Sample	Immune	RNA	Neurocognitive	Metal Ion	ATP

Arion BD 1 vs Control	Brain					
	
Arion BD NOS vs Control	Brain					

Bahn BD vs Control	Brain					

Bousman BD vs Control	Blood					

Clelland BD 1 vs Control	Blood					

Cruceanu BD vs Control	Brain					

Hilscher BD vs Control	Skin					
	
Iwamoto BD vs Control	Brain					
	
Kim BD 1 vs Control	Brain					

Logotheti BD 1 vs Control	Skin					

Matigian BD 1 vs Control	Blood					

Savitz BD vs Control	Blood					

Savitz BD 1 vs BD 2	Blood					

Vawter BD 1 vs Control	Brain					
	
Witt BD 1 vs Control	Blood					

**Table 5.** Most Frequent Hub Genes The list of hub genes from bulk, down-regulated, and up-regulated interaction networks who appear more than 2 times across the analyzed studies. These genes pertain to a number of functions in the immune system, the synapse, RNA processing, and cell signaling.

Gene	INS	FTSJ3	CD4	WDR36	KIAA0020	SST	SNAP25	SYT1	SPI1	PPARG	JUNB	DDX51
Count	5	4	4	3	3	3	3	3	3	3	3	3

## Data Availability

The data-sets supporting the conclusions of this article are included within the article and its additional files.
