# Supplementary material for: Immune, RNA, and Neurocognitive Genetic Networks in Bipolar Disorder Subtypes: A Transcriptomic Meta-Analysis
Source: Res Sq. 2024 Jan 17:rs.3.rs-3508951. Preprint. [Version 1] doi: 10.21203/rs.3.rs-3508951/v1 (PMC10836095; doi:10.21203/rs.3.rs-3508951/v1)
Supplement: 1 [file NIHPPrs3508951v1-supplement-1.pdf]

Supplementary Information

Supplementary Table 1

This table contains every differentially expressed gene identified by GEO2R analysis for each data set. This is categorized into differentially expressed genes with a p value < 0.5 and p value < 0.05 and LogFC $> |0.5|$. This file is an .xlsx file which can be viewed using Excel and other tools.

Supplementary Table 2

This table on sheet 1 contains every resulting GO term significantly enriched with an adjusted p value < 0.05. GO terms identified as immune, RNA, neurocognitive, metal ion, or ATP related are highlighted in green, red, orange, purple, and blue respectively. Sheet 2 contains which comparisons were enriched for each GO term pathway. Sheet 3 is a condensed summary of sheet 2. Sheet 4 describes the terms used in Excel to highlight each genetic pathway, as well as a list of terms that were excluded for more accurate analysis. This file is an .xlsx file which can be viewed using Excel and other tools.

Supplementary Table 3

This table contains the reported demographic information of the data sets analyzed in this study. This contains the number of samples, sex, race, source of material, diagnostic criteria, medication, and sequencing platform. This file is an .xlsx file which can be viewed using Excel and other tools.

Supplementary Table 4

This table contains the 10 hub genes of each data sets' bulk, down-regulated, and up-regulated differentially expressed gene analysis. Some data set comparisons contain less than 10 hub genes due to the small size of the data set, or a lack of connected proteins in the network. This file is an .xlsx file which can be viewed using Excel and other tools.

Supplementary Image 1-83

This folder contains 83 images generated in STRING or Cytoscape that depict the protein-protein interaction networks between DEGs found in each data set and condition, and with proteins of high confidence in the connecting network. This folder contains a sheet to explain which image contains what. Images are stored in PNG format.
